# Development of a Composite Pork Quality Index from Instrumental Traits and Its Hyperspectral Prediction

**DOI:** 10.3390/foods15162892

**Published:** 2026-08-18

**Authors:** Yiqi Rao, Yongzhe He, Siyao Huang, Qian You, Shuqi Tang, Hu Zhang, Liandong Luo, Xiaoyan Xu, Xingguo Tian

**Affiliations:** 1 The National Center for Precision Machining and Safety of Livestock and Poultry Products Joint Engineering Research Center, College of Food Science, South China Agricultural University, Guangzhou 510642, China; 2 Guangdong Provincial Key Laboratory of Food Quality and Safety, College of Food Science, South China Agricultural University, Guangzhou 510642, China; 3 College of Mathematics and Informatics, South China Agricultural University, Guangzhou 510642, China; 4 College of Engineering, South China Agricultural University, Guangzhou 510642, China

**Keywords:** pork quality, composite quality index, instrumental traits, machine learning, hyperspectral imaging

## Abstract

Pork quality is multidimensional, but conventional evaluation methods are usually destructive, time-consuming, and unsuitable for rapid grading. In this study, longissimus dorsi samples from pigs weighing 30–150 kg were used to construct a composite quality index (CQI) from instrumental traits. Traits related to color, water-holding capacity, antioxidant capacity, and texture were measured, and 13 traits were retained after principal component analysis. The resulting CQI represents a dataset-dependent summary of instrumental quality traits rather than a validated measure of sensory eating quality. Hyperspectral images were collected from pork slices, and CQI was predicted using interval combination optimization (ICO) combined with extreme gradient boosting (XGBoost). In the fixed spectral-level comparison, the SNV–ICO–XGBoost model achieved an *R*_p_^2^ of 0.8670, an *RMSE*_p_ of 1.7319, and an *RPD*_p_ of 2.7423. Animal-level mean-spectrum validation over 50 random splits showed that PLSR achieved the highest mean prediction performance, with an *R*_p_^2^ of 0.807 ± 0.058, an *RMSE*_p_ of 1.857 ± 0.345, and an *RPD*_p_ of 2.374 ± 0.345, providing a more conservative assessment of model performance. The predicted CQI values were further visualized qualitatively on the pork surface, supporting further evaluation of HSI for rapid and non-destructive assessment of instrumentally derived pork quality.

## 1. Introduction

Pork quality is not determined by a single trait. Color, water-holding capacity, texture, and antioxidant status all contribute to the instrumental quality profile of pork. These traits are influenced by genetics, postmortem biochemical changes, and environmental conditions, all of which can affect product quality and market value [1,2,3]. Traditional quality assessment relies on chemical analyses and sensory panels, which are destructive, labor-intensive, and ill-suited for rapid, real-time monitoring [4]. During postmortem storage, glycolysis and the accompanying pH decline occur together with myoglobin oxidation, protein degradation, and water migration. These changes are associated with variations in color, water-holding capacity, texture, and oxidative status. These postmortem changes may also be reflected in the optical properties of pork. Myoglobin changes can affect visible light absorption, while changes in water distribution and protein structure may influence the spectral response [5,6]. Because of this, a single measurement may not adequately describe the overall instrumental quality profile of pork [7]. There is therefore a clear need for a rapid, non-destructive method capable of evaluating pork quality across multiple dimensions.

Hyperspectral imaging (HSI) captures spatial images while collecting spectral information. This ability makes it well suited for non-destructive food quality assessment [8]. In pork, HSI was initially used to predict individual indicators like meat color and pH [4,9]. Subsequently, studies turned to analyzing multiple quality attributes at the same time. For example, Tang et al. [10] combined HSI with artificial neural networks to predict 14 physicochemical traits of pork and achieved spatial visualization of fat content. Xie et al. [11] further extended the application of HSI to the dynamic monitoring of the postmortem aging process using the wavelet transform. Machine learning models such as extreme gradient boosting and Transformers have further enhanced HSI’s ability to extract complex nonlinear information from spectra [12,13]. However, most existing studies still treat individual quality attributes as independent prediction targets, overlooking the intrinsic relationships among multidimensional pork quality traits [3,7]. Consequently, independent prediction of single indicators cannot directly provide a unified evaluation of pork quality.

Comprehensive evaluation models provide an effective strategy for integrating multidimensional information by assigning weights or reducing dimensionality across multiple indicators [14]. The entropy weight method assigns weights according to data variation [15], but it may not fully account for intrinsic correlations and directionality among variables [16]. Factor analysis can effectively reduce dimensionality and address multicollinearity and has been preliminarily applied in food quality evaluation [17]. Sun et al. [18] developed a comprehensive strawberry quality prediction model using HSI, and Dong et al. [19] combined factor analysis with LSTM to predict a comprehensive index of essential amino acids in beef. Those studies focused on fruit or specific nutritional traits of beef. In pork, meat color, water-holding capacity, texture, and antioxidant capacity do not work independently. These quality attributes are continuously influenced by postmortem biochemical processes. Consequently, most previous studies have focused on predicting individual quality traits, such as pH or meat color [10,11], or classifying pork into the predefined categories such as PSE and DFD [20]. Therefore, few studies have used HSI to predict a composite index derived from multiple instrumental pork-quality traits.

In this study, the longissimus dorsi samples from pigs in different pre-slaughter body-weight ranges were examined. Instrumental traits related to color, water-holding capacity, antioxidant capacity, and texture, were measured. PCA was used to construct a composite quality index (CQI) from the retained variables. Hyperspectral data were then collected, and machine learning models were used to predict CQI. Predicted CQI values were also mapped across the sample surface to illustrate their spatial distribution.

## 2. Materials and Methods

### 2.1. Experimental Materials

Pork longissimus dorsi samples were purchased from a commercial slaughterhouse in Shantou, China. The experimental animals were Large White × Landrace crossbred gilts. The supplier provided the pre-slaughter body weight and slaughter time corresponding to each sample. Based on the supplier-provided pre-slaughter body weight, the samples were divided into three groups: Group A (30 ≤ W < 70 kg), Group B (70 ≤ W < 110 kg), and Group C (110 ≤ W ≤ 150 kg). Each group contained 76 pigs, yielding 228 longissimus dorsi samples in total. One longissimus dorsi sample was collected from each pig. No other production or carcass records (age, diet, housing, transport, carcass weight, backfat, or intramuscular fat) were provided by the supplier.

Copper sulfate, sodium hydroxide, potassium chloride, potassium sodium tartrate, sodium dihydrogen phosphate, disodium hydrogen phosphate, sodium chloride, and bovine serum albumin were purchased from Guangzhou Chemical Reagent Factory (Guangzhou, China). EGTA and β-mercaptoethanol were obtained from Shanghai LiBo Chemical Technology Co., Ltd., Shanghai, China, T-SOD and MDA assay kits were purchased from Nanjing Jiancheng Bioengineering Institute (Nanjing, China), and the T-AOC assay kit was from Beijing Solarbio Science & Technology Co., Ltd., Beijing, China. All reagents were of analytical grade. The ethical review of this study was conducted in accordance with GB/T 35892-2018 [21], Laboratory animal—Guideline for ethical review of animal welfare [21] issued by the Ministry of Agriculture of China. The experimental protocol was approved by the Ethics Committee of South China Agricultural University (approval number: SCAU-2023G012). The pork samples were obtained from commercially slaughtered animals, and no live-animal procedures were performed by the research team.

### 2.2. Sample Preparation

Based on the supplier-provided slaughter times, the longissimus dorsi samples were kept at 4 °C until 24 h postmortem. Then, the samples were cut as specified in Table 1 for subsequent measurements.

### 2.3. Determination of Pork Quality Traits

#### 2.3.1. Meat Color and pH

A colorimeter was used for meat color (L*, a*, and b*). A 1 cm thick slice was cut parallel to the muscle fibers and trimmed to 5 × 5 × 1 cm. The instrument was calibrated with a standard white plate. Measurements were then taken at three positions using a CR-410 colorimeter (Konica Minolta, Tokyo, Japan).

pH was determined by direct insertion according to NY/T 821-2019 [22]. A calibrated pH meter with a temperature-compensation probe (Testo 205, Testo SE & Co. KGaA, Titisee-Neustadt, Germany) was inserted into the longissimus dorsi. The value was recorded after stabilization. Measurements were taken at three positions.

#### 2.3.2. Textural Properties

Shear force (SF) was measured according to the Chinese Agricultural Industry Standard NY/T 1180-2006 [23]. Three muscle blocks measuring 6 × 4 × 4 cm were prepared from each pig. The blocks were vacuum-sealed and heated in an 80 °C water bath until the core temperature reached 70 °C. Heating required approximately 20 min. After cooling to 0–4 °C, strips with a 1 × 1 cm cross-section and a minimum length of 2.5 cm were cut parallel to the muscle fibers. SF was measured using a TA. XT Plus texture analyzer (Stable Micro Systems, Godalming, UK) fitted with a WBS probe. The test speed was 2 mm/s.

Texture profile analysis (TPA) was performed according to He et al. [24]. Three muscle blocks from each pig were heated and cooled as described for SF. One 1.5 × 1 × 1 cm sample was cut from each block. Measurements were performed using the same texture analyzer fitted with a P/36R cylindrical probe. The pre-test, test, and post-test speeds were 1, 5, and 5 mm/s, respectively. The compression ratio was 50%, and the trigger force was 5 g. Hardness (HD), springiness (SP), cohesiveness (CO), gumminess (GM), chewiness (CH), and resilience (RE) were recorded. SF, HD, GM, and CH were expressed in gram-force, whereas SP, CO, and RE were dimensionless.

#### 2.3.3. Moisture Content and Water-Holding Properties

Moisture content (MC) was determined following the Chinese standard GB 5009.3-2016 [25]. Three minced subsamples were tested for each pig. Each subsample weighed 2–10 g and was no more than 5 mm thick. An aluminum box was dried to constant mass and weighed as *m*_0_. The sample was added and weighed as *m*_1_, dried at 101–105 °C for 2–4 h, cooled in a desiccator for 30 min, and reweighed. Drying was repeated until two successive measurements differed by less than 2 mg, and the final mass was recorded as *m*_2_. MC was calculated as follows:

(1) $$\mathrm{MC}\;=\;\frac{m_{1\;}-m_{2}}{m_{1}-m_{0}}\;\times \;100\%$$

where *m*_0_ is the mass of the empty aluminum box, *m*_1_ is the combined mass of the aluminum box and sample before drying, and *m*_2_ is the combined mass after drying to constant mass.

Water-holding capacity (WHC) was determined following the Chinese Agricultural Industry Standard NY/T 821-2019 [22]. Three cylindrical subsamples measuring 2.5 cm in diameter and 2 cm in height were prepared from each pig. Each subsample was weighed as *m*_1_, wrapped in two layers of 100-mesh gauze and one layer of qualitative filter paper, and placed in an RH-1000 water-holding capacity analyzer (Guangzhou Runhu Instrument Co., Ltd., Guangzhou, China). The instrument was operated at a load of 35 kgf, a test speed of 10 mm/min, a deformation ratio of 50%, and a test time of 300 s. After compression, the sample was removed and reweighed as *m*_2_. WHC was calculated as follows:

(2) $$\mathrm{WHC}\;=\;(1-\frac{m_{1\;}-m_{2}}{m_{1}\;\times \;\mathrm{MC}})\;\times \;100\%$$

where *m*_1_ is the sample mass before compression, *m*_2_ is the sample mass after compression, and MC is the moisture content determined by Equation (1), expressed as a decimal fraction for calculation.

Drip loss (DL) was determined according to NY/T 821-2019 [22]. Three strips measuring 2 × 3 × 5 cm were prepared from each pig after removal of the outer epimysium. Each strip was weighed as *m*_1_, suspended at 2–4 °C for 24 h without contacting the container wall or other samples, gently blotted dry, and reweighed as *m*_2_. DL was calculated as follows:

(3) $$\mathrm{DL}\;=\;\frac{m_{1\;}-m_{2}}{m_{1}}\;\times \;100\%$$

where *m*_1_ is the initial sample mass and *m*_2_ is the sample mass after 24 h of suspended storage.

Cooking loss (CL) was determined following Wan et al. [26], with slight modifications. Three 6 × 4 × 4 cm muscle blocks from each pig were weighed as *m*_1_, sealed in polyethylene bags, and heated in an 80 °C water bath until the core temperature reached 70 °C. Heating required approximately 20 min. The samples were cooled to 0–4 °C, gently blotted dry, and reweighed as *m*_2_. CL was calculated as follows:

(4) $$\mathrm{CL}\;=\;\frac{m_{1\;}-m_{2}}{m_{1}}\;\times \;100\%$$

where *m*_1_ is the sample mass before cooking and *m*_2_ is the sample mass after cooking and cooling.

#### 2.3.4. Antioxidant and Oxidative Status

Total superoxide dismutase (T-SOD) activity and malondialdehyde (MDA) content were measured using assay kits from Nanjing Jiancheng Bioengineering Institute, and total antioxidant capacity (T-AOC) was measured using a kit from Beijing Solarbio Science & Technology Co., Ltd., Beijing, China. Approximately 3 g of longissimus dorsi tissue from each pig was mixed with 27 mL of ice-cold 0.86% saline and homogenized twice in an ice bath at 10,000 rpm for 30 s using an FJ200-SH homogenizer (Shanghai Specimen Model Factory, Shanghai, China). The homogenate was filtered through two layers of 150-mesh gauze, and protein concentration was determined by the biuret method.

For T-SOD, 0.05 mL of homogenate was incubated with the kit reagents at 37 °C for 40 min. After color development and standing for 10 min, absorbance was measured at 550 nm using a UV-1800 spectrophotometer (Shimadzu, Kyoto, Japan). For MDA, 0.20 mL of homogenate was heated with the kit reagents at 95 °C for 80 min. After cooling, the mixture was centrifuged according to the kit instructions, and absorbance was measured at 532 nm. For T-AOC, 30 μL of homogenate was incubated with the kit reagents at room temperature for 10 min, and absorbance was measured at 593 nm. T-SOD and T-AOC were expressed as U/mg protein and MDA as nmol/mg protein.

Replicate measurements were averaged within each pig. Color and pH values were averaged from three positional readings. SF, TPA, MC, WHC, DL, and CL values were averaged from three tissue subsamples. T-SOD, MDA, and T-AOC values were averaged from three technical determinations of the homogenate. Subsequent statistical analyses used animal-level values. Each body-weight group contained 76 pigs.

### 2.4. Determination of Myofibrillar Protein Structure

Six pigs were randomly selected from each body-weight group for myofibrillar protein analysis. Myofibrillar proteins were extracted following Zhang et al. [27], and the myofibrillar fragmentation index (MFI) was determined following Zhang et al. [27]. Detailed procedures are provided in the Supplementary Materials.

### 2.5. Hyperspectral Data Acquisition and Extraction

**Figure 1:**
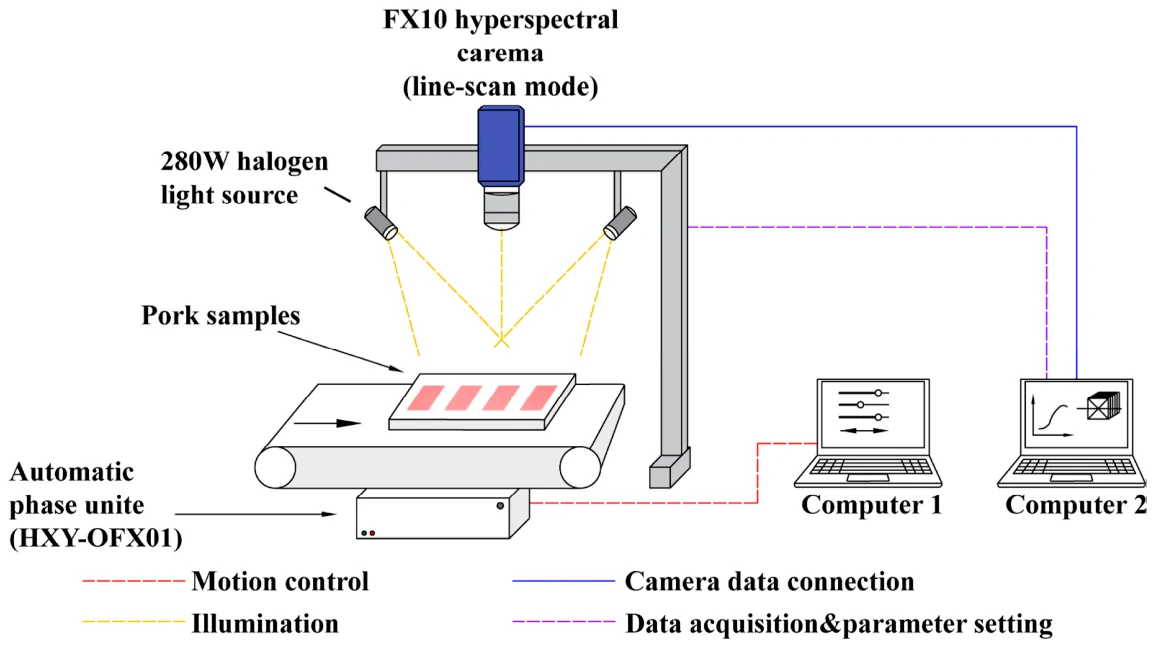
Schematic diagram of our laboratory’s hyperspectral imaging system. Note: The black arrow indicates the direction of sample movement, and the orange lines indicate illumination from the halogen light sources.

Hyperspectral images were acquired with a line-scanning system comprising an FX10 hyperspectral camera (SPECIM, Spectral Imaging Ltd., Oulu, Finland), a 280 W halogen light source, a motorized stage, and control computers (Figure 1). Three muscle tissue subsamples measuring 4 × 4 × 1 cm were prepared from each pig along the muscle fiber direction. After black-and-white correction [28], the images were converted to reflectance mode. The calibrated spectral range was 397.66–1003.81 nm, comprising 224 wavelength variables. The software (ENVI 4.8; ITT Visual Information Solutions, Boulder, CO, USA) was employed to extract the average spectral reflectance within the region of interest. One spectrum was extracted per subsample, yielding three per pig and 684 in total. The pig was the experimental unit. The three spectra were treated as subsample replicates, rather than independent observations. Detailed acquisition parameters and correction procedures are provided in the Supplementary Materials.

### 2.6. Comprehensive Score Calculation and Statistical Analysis

#### 2.6.1. Significance of Differences

Each pig was considered the experimental unit. Replicate measurements and subsample determinations from the same pig, as described in Section 2.3, were averaged to obtain one value for each trait. These measurements were not treated as independent observations. Before one-way ANOVA, ANOVA residuals were tested for normality using the Shapiro–Wilk test. Homogeneity of variances was assessed using Levene’s test. One-way ANOVA was performed with SPSS 27.0 to compare the three body-weight groups (*n* = 76 per group). Duncan’s multiple range test was used for multiple comparisons. Data is presented as mean ± standard deviation. The level of statistical significance was set at *p* < 0.05.

#### 2.6.2. Principal Component and Correlation Analyses

The animal-level quality data from 228 pigs were examined for suitability for PCA using the KMO measure and Bartlett’s sphericity test. PCA was performed with the FACTOR procedure in SPSS 27.0, with principal components extracted from the correlation matrix. An extraction communality of 0.50 was used as the cutoff for variable screening. Variables below this threshold were removed. Thus, at least half of the variance in each retained variable was explained by the component solution. PCA was repeated after screening. The eigenvalue-greater-than-one criterion guided component retention, while the scree plot provided additional support for the final decision. Varimax rotation was applied to aid interpretation of the resulting component structure.

Correlations among the composite indices and selected quality traits were visualized using a scatter plot matrix. Pearson correlation coefficients were calculated, with asterisks (*) denoting *p* < 0.05. The analysis was performed using Python 3.13.

#### 2.6.3. Construction of Composite Indices

The 18 traits had no missing values in the 228 pigs, so no imputation or deletion was needed. Before calculating the index, each retained trait was rescaled across all animals by min–max normalization:

(5) $$\;x_{\mathrm{ij}}^{*}=0.001+\frac{x_{\mathrm{ij}}-\min(x_{j})}{\max(x_{j})-\min(x_{j})}$$

where *x*_ij_ denotes the original value of trait j for pig i. The constant 0.001 was added to keep all normalized values above zero for subsequent multiplication, division, and logarithmic transformation. Normalization and CQI calculation were completed before spectral dataset partitioning.

Based on the rotated factor-loading matrix described in Section 2.6.2 [17], the retained traits were grouped by the signs of their PC1 loadings. The grouping determined only which traits went to the numerator or denominator. It was not interpreted as a biological effect. Accordingly, the CQI for each pig was calculated as:

(6) $$\mathrm{CQI}\;=\;K\;\times \;\ln\left[\frac{\Psi_{1}\;\times \;{\Psi \;}_{2\;}{\;\times \ldots \times \;\Psi }_{p}}{\Phi_{1}\;\times \;\Phi_{2}\;\times \;\ldots \times {\;\Phi }_{q}}\right]$$

where K was set to 1000, Ψ denotes a normalized trait in the positive-loading group, and *Φ* represents a normalized trait in the negative-loading group. No absolute-value transformation was applied because the 0.001 offset ensured that all normalized inputs to the CQI were strictly positive. The constant K changed only the numerical scale of the CQI and did not affect the ranking of the pigs. Each retained trait entered Equation (6) once, equivalent to an exponent of one. Equal exponents were used because no independent biological or sensory basis was available for assigning unequal weights; this mathematical treatment does not imply that all retained traits have equal biological importance. The multiplicative ratio was used to combine the relative variation in the two groups of normalized traits, while the logarithmic transformation reduced the numerical range of the resulting index. 

For comparison, a comprehensive weight index (CWI) was calculated using the component score coefficients and variance contribution rates of the five rotated components. For each retained trait, the overall scoring coefficient was calculated as:

(7) $$\;c\;=\;\beta_{1}v_{1}\;+{\;\beta }_{2}v_{2}\;+\;\beta_{3}v_{3}\;+\;\beta_{4}v_{4}\;+{\;\beta }_{5}v_{5}$$

where *β*_1_–*β*_5_ are the component score coefficients of the trait for the five rotated components, and *v*_1_–*v*_5_ are the corresponding variance contribution rates expressed as proportions. The coefficient for each trait was normalized as:

(8) $$\;W\;=\;\frac{c}{c_{1}\;+\;c_{2}\;+\;...\;+{\;c}_{m}}$$

where *c* is the overall coefficient of the trait and *c*_1_–*c*_m_ are the overall coefficients of all retained traits. The signs of the coefficients were retained, and no absolute-value transformation was applied. Before CWI calculation, each retained trait was min–max normalized without the 0.001 offset:

(9) $$\;u\;=\;\frac{x\;-\;\min(x)}{\max(x)\;-\;\min(x)}$$

where *x* is the original trait value for a given pig, and min(*x*) and max(*x*) are the minimum and maximum values of that trait among all 228 pigs. A CWI value was calculated separately for each pig as:

(10) $$\;\mathrm{CWI}\;=\sum_{j=1}^{n}\left(u_{\mathrm{ij}}\;\times \;W_{j}\right)$$

where *u*_ij_ represents the standardized value of the *j*th indicator for the ith sample, and *W*_j_ denotes the normalized scoring coefficient of the *j*th indicator.

### 2.7. Hyperspectral Data Preprocessing and Dataset Partitioning

The corrected spectra comprised 224 wavelength bands spanning 397.66–1003.81 nm. The spectra were preprocessed using Standard Normal Variate (SNV) transformation, Savitzky–Golay (SG) smoothing, multiplicative scatter correction (MSC), and baseline correction. SNV and MSC were applied to handle scattering effects caused by uneven sample surfaces [29,30]. SG smoothing and baseline correction were used to suppress spectral noise and correct background drift [31,32].

SNV was applied independently to each spectrum as follows:

(11) $$\;x_{j}^{\prime }\;=\;\frac{x_{j}-\bar{x}}{s}$$

where *x*_j_ is the reflectance at wavelength j, $\bar{x}$ is the mean reflectance across the 224 wavelengths, and *s* indicates the corresponding standard deviation. Spectral preprocessing was performed using The Unscrambler X 10.4. The settings and procedures for SG smoothing, MSC, and baseline correction are provided in Supplementary Materials.

The dataset was split into calibration and prediction subsets at a 3:1 ratio using the Sample Set Partitioning based on Joint X-Y Distances (SPXY) algorithm [33]. Partitioning was conducted at the animal level. For SPXY, the three replicate spectra from each pig were averaged to obtain one spectrum per pig. The averaged spectra formed the *X* matrix, and the CQI values formed the *Y* vector. Before partitioning, the animal records were randomly shuffled using a seed of 42. The 228 pigs were divided into 171 calibration animals and 57 prediction animals. Animal membership was then mapped back to the replicate spectra, ensuring that all three spectra from the same pig were assigned to the same subset. The calibration and prediction subsets contained 513 and 171 spectra. All partitioning was performed in MATLAB R2022a.

### 2.8. Hyperspectral Wavelength Selection

To reduce data redundancy and optimize model structure, four wavelength-selection methods were applied to the SNV-pretreated calibration spectra: interval combination optimization (ICO), variable iterative space shrinkage approach (VISSA), competitive adaptive reweighted sampling (CARS), and uninformative variable elimination (UVE). ICO divides the spectra into equal-width intervals and searches for the optimal interval combination through model population analysis [34]. VISSA dynamically shrinks the search space in each iteration, progressively eliminating unimportant wavebands [35]. CARS retains important variables through adaptive reweighting and exponentially decreasing force [36]. UVE introduces random noise variables as references and eliminates variables with low stability [37].

CARS used 50 Monte Carlo runs, with 80% of the calibration samples included in each run. VISSA used 39 iterations, with 500 weighted binary submodels per iteration and an 80% sample ratio. The procedure stopped after five consecutive iterations without improvement. UVE introduced 224 artificial noise variables and repeated noise generation 100 times. For ICO, the spectrum was divided into 40 initial intervals, each containing approximately 5–6 adjacent wavelengths. ICO evaluated 100 submodels per iteration, with a maximum of 50 iterations. A random seed of 42 was used for all procedures. Candidate wavelength subsets generated by CARS, VISSA, and ICO were evaluated using PLSR with five-fold cross-validation on the calibration subset. The subset with the lowest root mean square error in cross-validation was retained. For UVE, wavelengths were retained when their stability values exceeded the maximum absolute stability value of the artificial noise variables. The associated PLSR analysis used leave-one-out cross-validation.

The prediction subset was not used for wavelength selection. Detailed settings and the retained wavelengths for each method are given in Tables S1 and S2. All wavelength-selection procedures were performed in MATLAB R2022a.

### 2.9. Model Construction and Evaluation

#### 2.9.1. Model Construction

To establish a quantitative relationship between input variables and CQI, three regression models were compared: partial least squares regression (PLSR), backpropagation neural network (BPNN), and extreme gradient boosting (XGBoost). PLSR develops a linear model handling high-dimensional, multicollinear data for stable prediction [38]. BPNN uses multiple layers and adjusts its weights via backpropagation and gradient descent, which makes it well-suited for capturing complex nonlinear patterns [39]. XGBoost, a gradient-boosted tree algorithm, employs regularization and parallel computation to capture both linear and nonlinear relationships [40].

For the comparison of spectral preprocessing methods, PLSR models with 1–30 latent variables were evaluated using five-fold cross-validation within the calibration subset. The number of latent variables yielding the lowest root mean square error in cross-validation was selected. Each PLSR model was then refitted using the corresponding calibration spectra. For the comparison of wavelength-selection methods, 1–20 latent variables were evaluated using the same five-fold cross-validation procedure. The calibration-set cross-validation results were used only to select the number of latent variables for each input.

The spectral-level BPNN contained two hidden layers with 64 and 32 neurons, respectively. The hyperbolic tangent function was used as the activation function. Model parameters were updated using the Adam optimizer. The learning rate was 0.001, the L2 regularization coefficient was 0.0001, and the maximum number of training iterations was 2000. Ten percent of the calibration spectra were used for internal validation. Early stopping was applied with a patience of 20 iterations. The random seed was set to 42. These settings were kept constant across the wavelength-subsets. For the fixed-partition analysis, the BPNN was trained once using a single initialization. The PLSR and BPNN settings are provided in Table S3.

XGBoost was fitted using the squared-error objective function. Hyperparameters were optimized separately for the CARS, VISSA, UVE, and ICO wavelength subsets through randomized search. For each subset, 50 parameter combinations were evaluated using five-fold cross-validation within the calibration subset. The combination yielding the lowest mean root mean square error across the five folds was selected. The search space included the number of estimators, learning rate, maximum tree depth, minimum child weight, subsampling ratio, column-sampling ratio, gamma, L1 regularization, and L2 regularization. The random seed was set to 42. After hyperparameter selection, each model was full using the entire calibration subset and evaluated on the fixed prediction subset. The XGBoost search space and the selected parameters are provided in Table S4.

Wavelength selection, latent-variable selection, and hyperparameter optimization were conducted using only the calibration subset. The same prediction subset was then used to compare preprocessing methods, wavelength subsets, and regression models. It was treated as an internal comparison set rather than an external validation set. The Unscrambler X 10.4 was used for spectral preprocessing. MATLAB R2022a was used for dataset partitioning, wavelength selection, and fixed-partition spectral-level modeling. The repeated animal-level analysis was conducted using Python 3.13.

#### 2.9.2. Pixel-Wise Prediction of CQI

After background removal, using a binary mask generated from the difference image between bands 212 and 93 with a segmentation threshold of 0.03, the spectra of all sample pixels were processed using the same procedures and wavelength subset used for model development. Pixels within the binary sample mask, including those along the sample boundary, were retained for prediction. No additional illumination-quality threshold or edge-pixel exclusion was applied. A formal pixel-level applicability-domain assessment against the calibration spectra was not performed. The selected model was then applied pixel-wise. The predicted CQI values were not clipped to the range of the calibration subset. They were displayed as color maps using a common color scale across all samples. The color limits were set to the overall minimum and maximum predicted CQI values across the displayed samples. Because pixel-level reference CQI measurements were unavailable, the maps were interpreted qualitatively. All analyses were performed in MATLAB R2022a.

#### 2.9.3. Model Evaluation and Animal-Level Robustness Assessment

This study evaluates model performance using the coefficient of determination (*R*^2^), root mean square error (*RMSE*), and relative percentage deviation (*RPD*). The formulas are shown in Equations (12)–(14).

(12) $${\;R}^{2}=1\;-\;\frac{{\sum_{1}^{n}\left(\bar{y}-y_{i,p}\right)}^{2}}{{\sum_{1}^{n}\left(y_{i}-\bar{y}\right)}^{2}}$$

(13) $$RMSE\;=\sqrt{\frac{{\sum_{1}^{n}\left(y_{i,p}-y_{i}\right)}^{2}}{n}}$$

(14) $$RPD\;=\frac{SD}{{RMSE}_{P}}$$

where *y*_i,p_ is the predicted value, *y*_i_ denotes the true value, $\bar{y}$ indicates the mean of the true values, n is the sample size, SD represents the standard deviation of the true values in the prediction subset, and *RMSE*_p_ is the root mean square error of the test set.

Because three replicate spectra were obtained from each animal, animal-level validation was conducted using mean spectra. For each animal, the three spectra were averaged wavelength by wavelength, yielding one spectrum per pig. All 224 wavelengths were retained. This produced 228 animal-level observations. The resulting dataset was randomly divided into calibration and prediction subsets at a 3:1 ratio. This partitioning was repeated 50 times using random seeds from 0 to 49. Each partition included 171 calibration pigs and 57 prediction pigs.

In each partition, PLSR, BPNN, and XGBoost models were established. For PLSR, 1–20 latent variables were evaluated using five-fold cross-validation within the calibration subset. The number with the lowest RMSE was selected. BPNN and XGBoost were refitted using the fixed settings listed in Table S5. Wavelength selection and hyperparameter optimization were not repeated. *R*_c_^2^, *RMSE*_C_, *R*_p_^2^, *RMSE*_P_ and *RPD*_p_ were calculated. The results over the 50 partitions were summarized as mean ± standard deviation.

## 3. Results and Discussion

### 3.1. Differences in Instrumental Pork Quality Traits Across Body Weight Groups

To compare instrumental pork quality traits among body-weight groups, eighteen indicators related to meat color, water-holding capacity, textural properties, and antioxidant capacity were determined in the longissimus dorsi muscles from Groups A, B, and C. The corresponding results are summarized in Table 2.

For meat color, the L*, a*, and b* values were highest in Group B, followed by Group C, and lowest in Group A. A higher a* indicates greater redness, an important visual attribute of fresh pork [41]. Group B therefore had the highest instrumental redness.

The antioxidant indicators showed a different pattern. Group A showed the highest T-SOD activity. The redox state of myoglobin affects meat color [5]. However, antioxidant capacity alone could not explain the lower a* value in Group A. Differences in myoglobin state and water status may also have contributed. Because myoglobin forms were not measured, this interpretation remains tentative.

MC decreased with increasing pre-slaughter body weight, while WHC showed the opposite trend. Groups B and C had significantly higher WHC than Group A. Since WHC is affected by pH and the structural state of myofibrillar proteins [6], pH and protein-related indicators were considered. Although pH differed among the groups, the values were close and did not follow the same pattern as WHC. The differences in WHC may be more closely related to myofibrillar protein structure than to pH.

In terms of textural properties, SF, HD, and GM were generally higher in the heavier groups, but SP and CH showed less consistent patterns. Textural properties are closely associated with the structural integrity of myofibrillar proteins [42]. The higher SF and HD values in Groups B and C indicate greater resistance during instrumental testing, but not necessarily greater sensory toughness or chewiness. MFI represents the proportion of myofibrillar fragments between 1 and 4 sarcomeres in length relative to the total number of fragments, with a higher MFI indicating greater disruption of the internal myofibrillar structure [27]. As shown in Table S6, Group A had a significantly higher MFI than Groups B and C, consistent with greater myofibrillar fragmentation in Group A. Higher proportions of *α*-helix and β-sheet structures are generally associated with greater protein stability [43]. As shown in Table S7 and Figure S1, the proportions of *β*-turn and random coil were highest in Group A, whereas Group C had the highest *α*-helix content and Group B the highest *β*-sheet content. This pattern was consistent with greater disruption of ordered secondary structure in Group A during postmortem aging. Although the myofibrillar protein structure of Group B was relatively stable, some *α*-helices were still hydrolyzed and converted into *β*-sheets. In contrast, the myofibrillar protein structure of Group C was the most stable and underwent the least hydrolysis. As shown in Figure S2, with decreasing pre-slaughter body weight, the fluorescence peaks of tryptophan at 330 nm and phenylalanine at 295 nm gradually blue-shifted, accompanied by a significant decrease in intensity. Intrinsic fluorescence is sensitive to changes in the microenvironment of aromatic amino acid residues. The observed blue shift and decrease in fluorescence intensity indicate changes in the microenvironment of aromatic residues and alterations in tertiary structure [44]. This was consistent with the circular dichroism results. The fluorescence results were consistent with differences in protein conformation among the groups. However, the specific molecular factors contributing to the observed fluorescence-intensity differences could not be determined from the present data.

Protein structural stability is mainly maintained by non-covalent interactions, including hydrogen bonds, ionic bonds, and hydrophobic interactions [44]. To further characterize the structural differences among the three groups on myofibrillar protein structure, the chemical forces in the three groups were measured, with results shown in Table S6. Ionic bonds contribute to the structural stability of myofibrillar proteins through electrostatic interactions [45]. The ionic bond content was similar in Groups C and B and higher than that in Group A. In contrast, disulfide bonds showed a trend of Group C > Group B > Group A. Disulfide bonds enhance the stability of the protein tertiary structure by reducing the conformational entropy of the denatured state [46] and help immobilize water molecules, thereby improving the water-holding capacity of meat [6]. The higher disulfide bond content in Groups B and C was consistent with their higher WHC, which may provide a possible structural explanation for the differences in water-holding capacity. The hydrophobic interaction in Group A was significantly higher than in Groups B and C. Destabilization of protein structure may promote hydrophobic group exposure and protein aggregation [27], which may account for the less stable tertiary structure in Group A. Hydrogen bond content increased with increasing pre-slaughter body weight, consistent with the trend in *α*-helix content [43]. Therefore, the myofibrillar protein results were consistent with the group differences in water-holding capacity and texture. They did not establish a direct effect of pre-slaughter body weight.

In summary, the three groups differed across the measured instrumental quality traits, but no single pattern emerged with increasing body weight. This made it difficult to summarize the instrumental quality profile using a single indicator and supported combining the traits into a composite index. The pigs were grouped by observed pre-slaughter body weight rather than assigned to an experimental treatment. Information on age, growth rate, feeding history, and production batch was unavailable. These factors may have contributed to the group differences. The findings should be interpreted as associations with body-weight group, not as direct effects of body weight.

### 3.2. Variable Screening and Principal Component Analysis

Principal component analysis was performed on the 18 indicators to examine the covariance structure of instrumental pork-quality traits across different groups. In Table S8, the KMO measure was 0.706, above the usual 0.7 threshold. Bartlett’s test of sphericity came out highly significant (*p* < 0.001), showing that the data were suitable for PCA [47]. Five indicators fell below the 0.5 extraction cutoff during communality screening (pH, DL, CL, SP, and MDA) and were dropped. The remaining 13 all exceeded this threshold (Table S9). The first five principal components each had an eigenvalue above 1, explaining 81.678% of the total variance (Table 3). The scree plot further supported the retention of five principal components (Figure S3). Table 3 summarizes the eigenvalues and variance contributions of the extracted principal components. PC1 mainly captured a*, b*, MC, WHC, SF, HD, CH, and GM. PC2 was mainly related to CO, GM, and RE. PC3 reflected L*, b*, T-AOC, and T-SOD, while PC4 captured L*, b*, T-AOC, T-SOD, WHC, and SF. PC5 mainly reflected MC and WHC. Together, these five components summarized the main dimensions of the instrumental pork-quality profile [17].

### 3.3. Construction and Comparison of CQI and CWI

#### 3.3.1. Construction of CQI and CWI

Based on the PCA results, the rotated component matrix was further examined to determine the contribution direction of each indicator. L*, a*, b*, WHC, SF, HD, GM, and CH loaded on the positive side, while T-AOC, T-SOD, MC, CO, and RE fell on the negative. This classification was based on the sign of each indicator’s loading on PC1 (Table S10). The sign was used only to determine whether each indicator entered the numerator or denominator of the CQI equation. It was not interpreted as a favorable or unfavorable biological direction. After normalization, CQI was computed for each group using Equation (15) [17]. For comparison, CWI was calculated from comprehensive scoring coefficients derived from the component score coefficients and the variance contribution of the five retained components (Table S11). The standardized data were then combined with these coefficients using Equation (16). The CQI and CWI values for the three groups are presented in Table 4.

(15) $$\;CQI=1000\;\times \;\ln\left[\frac{\Psi_{L*}\;\times {\;\Psi }_{a*}{\;\times {\;\Psi }_{b*}\;\times {\;\Psi }_{\mathrm{WHC}}\;\times {\;\Psi }_{\mathrm{SF}}\;\times \;\Psi_{HD}\;\times {\;\Psi }_{\mathrm{GM}}\;\times \;\Psi }_{\mathrm{CH}}}{\Phi_{T-\mathrm{AOC}}\;\times \;\Phi_{T-\mathrm{SOD}}\;\times {\;\Phi }_{\mathrm{MC}}\;\times {\;\Phi }_{CO}\;\times {\;\Phi }_{\mathrm{RE}}}\right]$$

(16) $$\;CWI=\left(\begin{array}{c} 7.36\%u_{i1}+6.24\%u_{i2}+8.68{\%u}_{i3}+10.31{\%u}_{i4}+10.91{\%u}_{i5}\\ -6.67{\%u}_{i6}+6.96{\%u}_{i7}+4.28{\%u}_{i8}+2.21{\%u}_{i9}+10.95{\%u}_{i10}\\ +10.89{\%u}_{i11}+10.26{\%u}_{i12}+13.37{\%u}_{i13} \end{array}\right)$$

Across all 228 pigs, the animal-level CQI values had a mean of 4.389 ± 5.258, a median of 6.211, an interquartile range of 0.280–7.855, and a range from −12.042 to 16.706. The Fisher–Pearson sample skewness was −0.940, indicating a moderately left-skewed distribution. Based on Tukey’s 1.5 × interquartile-range rule, one observation fell below the lower Tukey limit and was identified as a potential extreme observation. This observation was retained because univariate distributional screening alone was not used as a criterion for data exclusion.

Because CQI is expressed as 1000 times the natural logarithm of a product ratio, a one-unit difference corresponds to a 0.1% increase in the underlying ratio (exp (0.001) ≈ 1.001). This small proportional change does not represent a fixed one-unit improvement in biological, sensory, or consumer-perceived pork quality. CQI values should therefore be interpreted only as relative positions on a dataset-dependent instrumental index.

The CQI derived from all 18 traits was strongly correlated with the 13-trait CQI (Spearman’s ρ = 0.959, *p* < 0.001).

#### 3.3.2. Comparison of CQI and CWI

**Figure 2:**
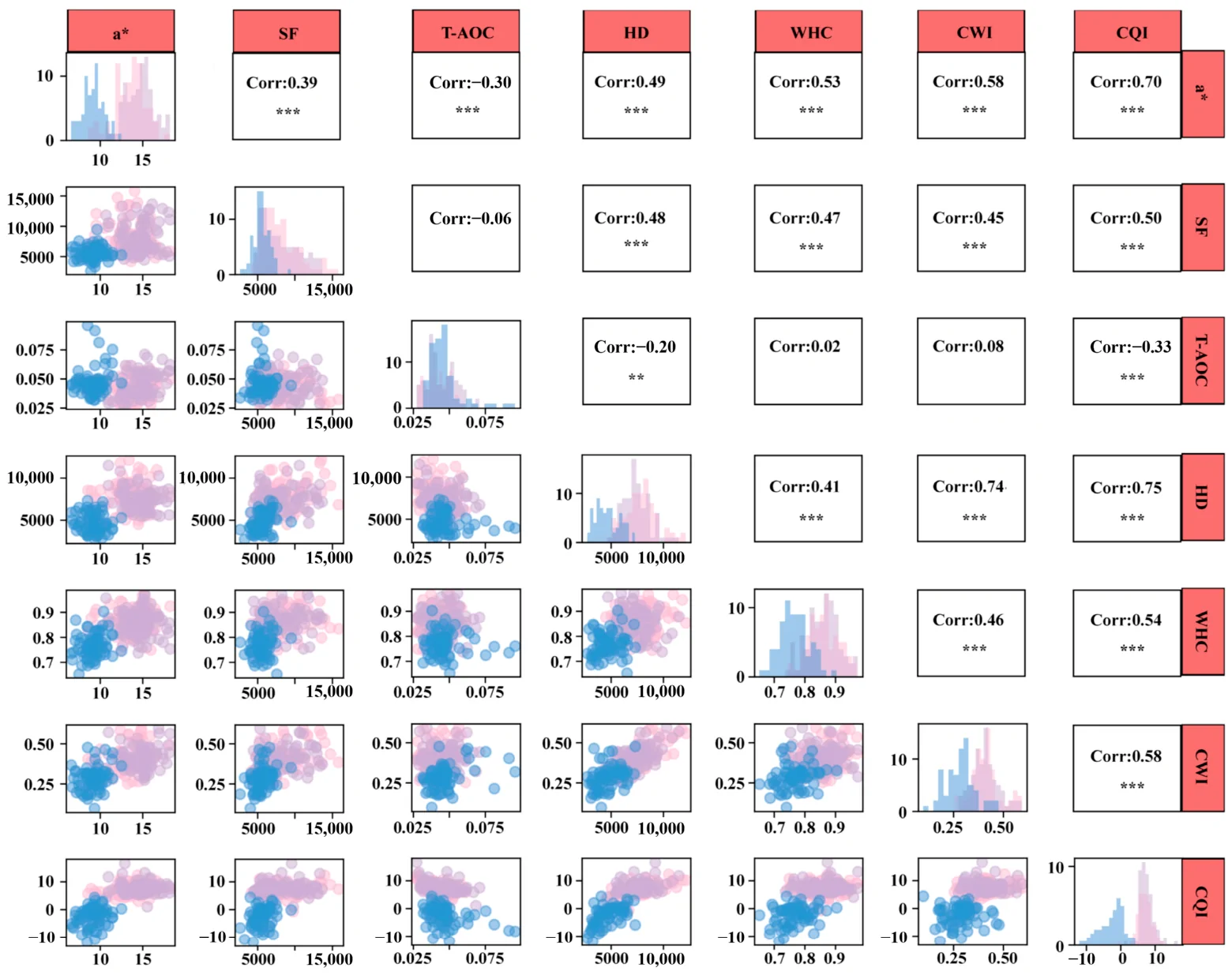
Scatter plot of multivariate correlations. Blue denotes Group A (30–70 kg), pink denotes Group B (70–110 kg), and purple denotes Group C (110–150 kg). “*”: *p* < 0.05, “**”: *p* < 0.01, “***”: *p* < 0.001.

To compare CQI and CWI, a multivariate correlation analysis was performed among the two indices (CQI and CWI) and five representative indicators: a* (appearance), T-AOC (antioxidant capacity), WHC (water-holding capacity), SF, and HD (textural properties). As shown in Figure 2, CQI was significantly correlated with a* (r = 0.70, 95% CI: 0.63–0.76), SF (r = 0.50, 95% CI: 0.40–0.59), T-AOC (r = −0.33, 95% CI: −0.44 to −0.21), HD (r = 0.75, 95% CI: 0.69–0.80), and WHC (r = 0.54, 95% CI: 0.44–0.63; all *p* < 0.001). CWI was significantly correlated with a*, SF, HD, and WHC (r = 0.45–0.74, all *p* < 0.001), but not with T-AOC (r = 0.08, 95% CI: −0.05–0.21, *p* = 0.229; n = 228). Because these traits were included in the construction of the indices, the correlations represent internal associations rather than independent validation.

The weaker explanatory power of CWI for antioxidant indicators may be related to the construction of composite coefficients. In CWI, the component score coefficients of each trait were combined across the retained principal components according to their variance contributions. Consequently, traits that loaded mainly on components with smaller variance contributions had less influence on the final index. Antioxidant traits are closely associated with meat color and water-holding capacity. For example, T-AOC and T-SOD affect the myoglobin redox state and thus color stability [5], while oxidation of myofibrillar proteins can induce conformational changes and weaken protein–water interactions [6]. When such interdependent attributes are integrated into a single weighted score, the contribution of antioxidant indicators may be partly diluted. In contrast, CQI grouped indicators according to the direction of their loadings, which may explain its broader associations with the selected instrumental traits in the present dataset.

Based on its broader associations with the selected instrumental traits in the present dataset, CQI was retained as the target for subsequent modeling.

### 3.4. Spectral Analysis of Samples Across Different Groups

**Figure 3:**
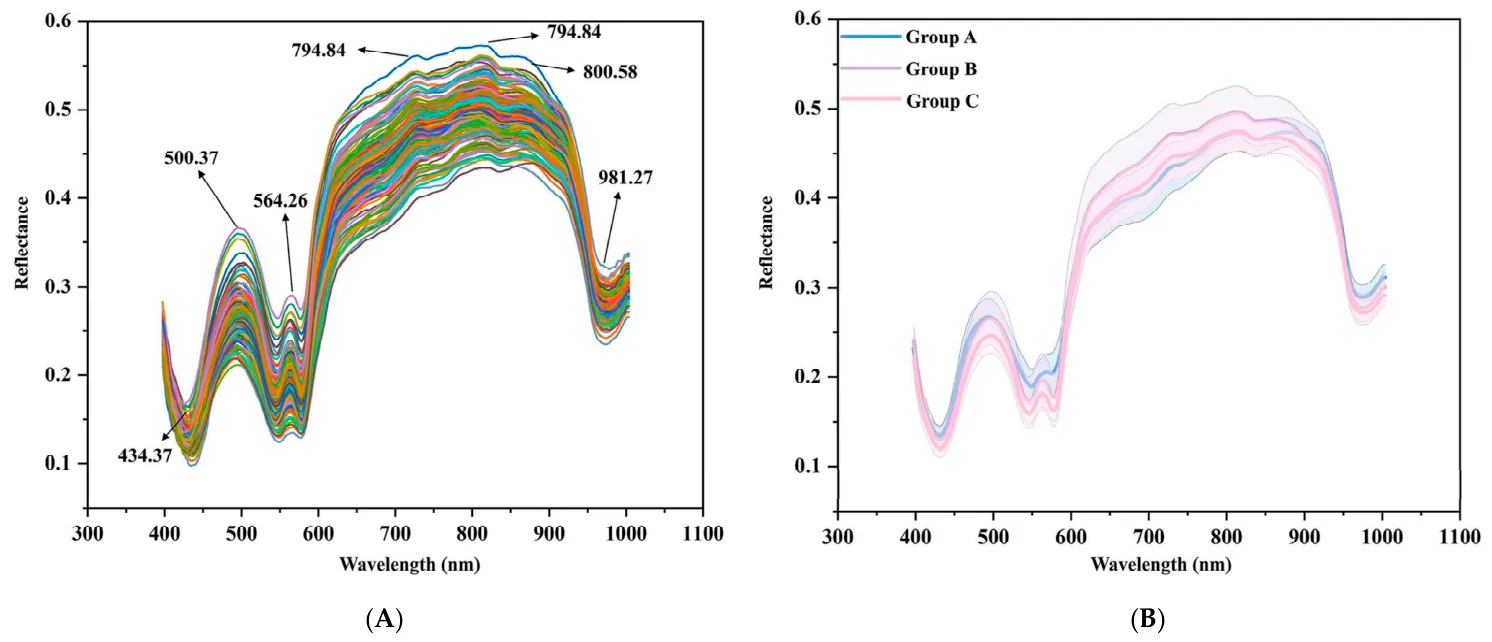
Raw and average spectral curves of pork samples from Groups A, B, and C in the 397.66–1003.81 nm range: (**A**) raw spectral curves; (**B**) average spectral curves.

The original and average reflectance spectra of all samples across the 397.66–1003.81 nm range are shown in Figure 3A,B. Although the spectral profiles were generally similar, differences among groups could still be observed at several characteristic wavelengths. Clear spectral features appeared near 434.37, 500.37, 564.26, 794.84, 880.58, and 981.27 nm. The spectra of Groups B and C were relatively close to each other, whereas Group A showed more obvious differences, especially around 564.26, 880.58, and 981.27 nm. These features were interpreted cautiously because the spectral bands are broad and may contain overlapping chemical and physical information.

Most of the visible-region features were associated with myoglobin. The valley around 434.37 nm was associated with the Soret band [48], while the features near 500.37 and 564.26 nm may be related to differences among myoglobin derivatives [49]. The difference around 564.26 nm was consistent with the lower a^*^ value of Group A. Because the absorption bands of different myoglobin forms overlap, this variation cannot be attributed to oxymyoglobin alone.

In the NIR region, the spectral differences may reflect variations in water status and protein structure. The feature near 794.84 nm and the valley near 981.27 nm have been associated with O–H overtones of water [50]. The clear difference around 981.27 nm was consistent with the lower water-holding capacity of Group A. A similar difference was also observed around 880.58 nm, which may be related to the third overtone of protein N–H stretching [4]. However, these spectral assignments are tentative because VIS–NIR bands are broad and overlapping and may also be influenced by tissue structure and light scattering. Weaker responses near 720 and 850 nm were also found in Group A. These regions have been linked to the related tenderness properties [51], and this trend was consistent with the higher MFI and lower shear force and hardness values of Group A.

Overall, the spectral differences among the three groups were consistent with combined variation in meat color, water status, and protein structure. These differences supported subsequent CQI prediction modelling.

### 3.5. Comparison of Spectral Pretreatment Methods

**Figure 4:**
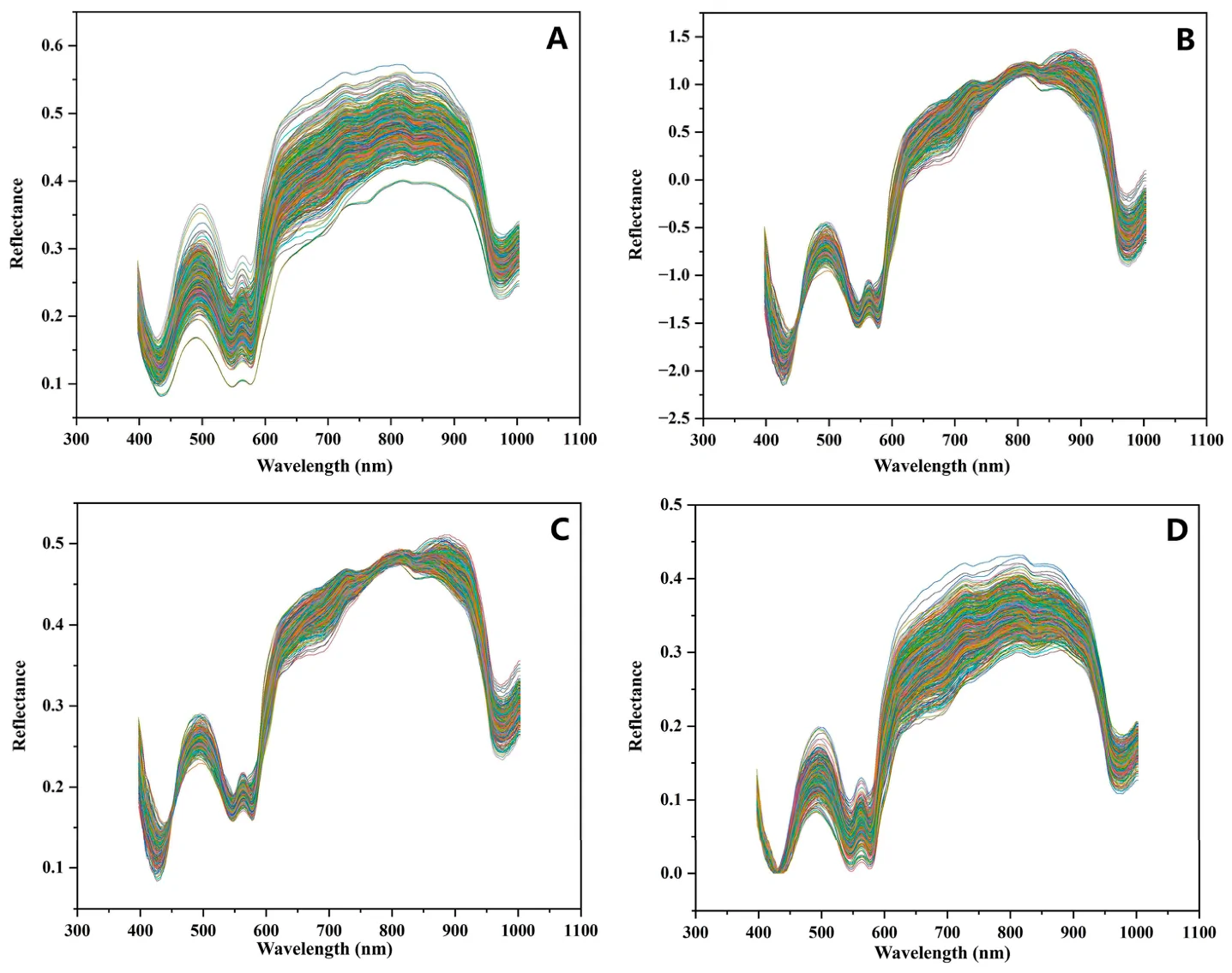
Preprocessed spectral curves of pork samples from Groups A, B, and C: (**A**) SG smoothing; (**B**) SNV; (**C**) MSC; (**D**) Baseline correction. Note: Different colors represent individual sample spectra and are used only for visual distinction.

Figure 4 shows the spectra obtained after different preprocessing treatments. SG smoothing mainly reduced random noise and made the curves smoother. SNV and MSC were applied to reduce scattering effects caused by uneven sample surfaces, whereas baseline correction was used to adjust background drift [31]. PLSR models were built for each preprocessing method, and the results are shown in Table 5.

Model performance among the preprocessing methods (Table 5). Uneven sample surfaces can cause incident light to scatter to varying degrees, obscuring spectral responses related to myoglobin, water, and protein components. Both SNV and MSC improved model performance compared with the original spectra. SNV normalizes each spectrum independently, eliminating multiplicative and additive scattering effects [29], whereas MSC performs correction using the mean spectrum as a reference [30]. MSC-PLSR achieved a higher R_c_^2^ and a lower RMSE_C_, indicating a better calibration fit. In contrast, SNV-PLSR yielded a lower RMSE_P_ and a slightly higher R_p_^2^. Because the aim was to predict CQI rather than maximize calibration fit, the preprocessing method was selected based on prediction-subset performance. SNV-preprocessed spectra were therefore used in the subsequent wavelength-selection and modeling analyses.

### 3.6. Spectral Feature Wavelength Selection

**Figure 5:**
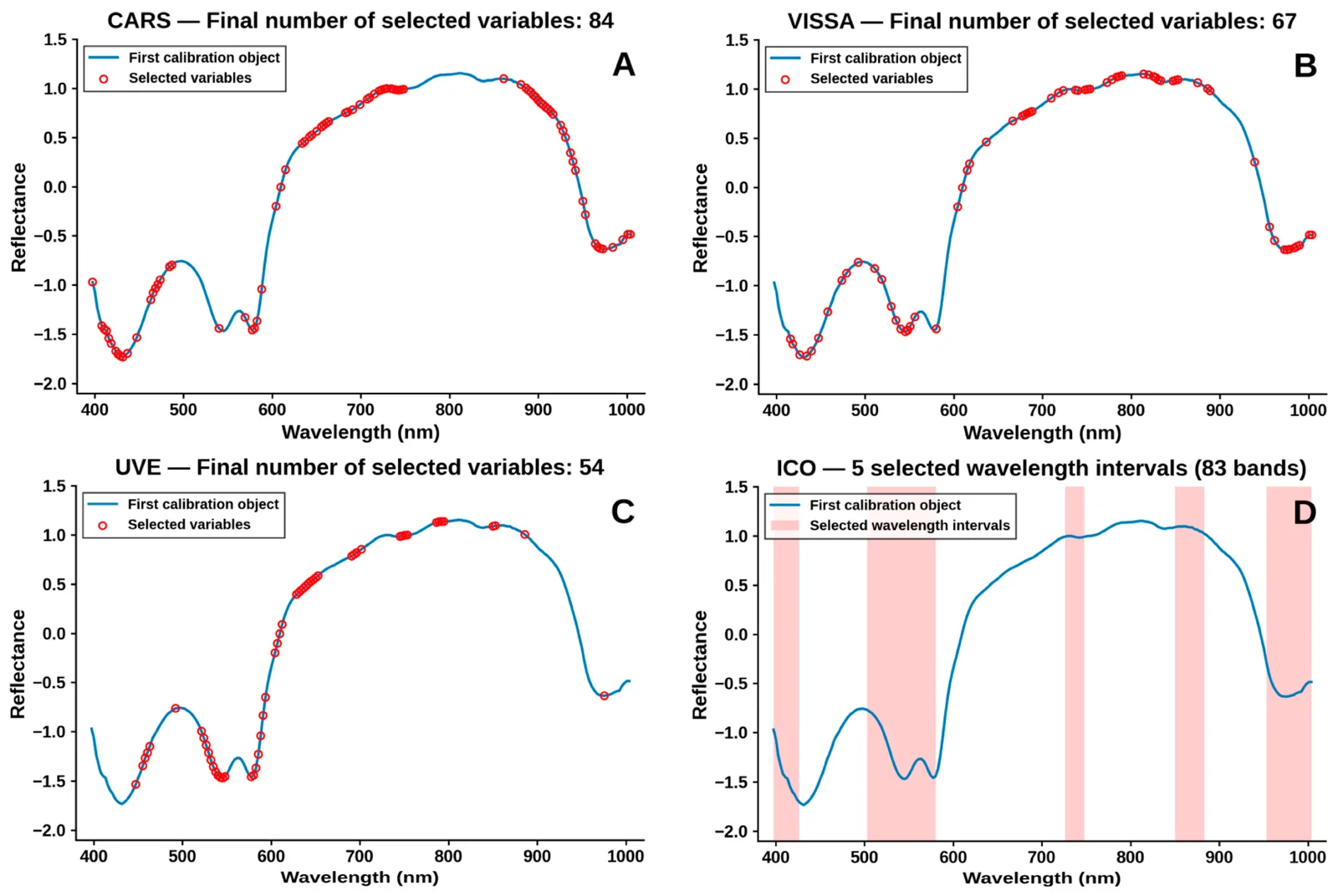
Characteristic wavelength selection for CQI using different feature selection methods. (**A**) CARS; (**B**) VISSA; (**C**) UVE; (**D**) ICO.

The hyperspectral data contained 224 wavebands, of which only part carried useful information related to CQI. Therefore, CARS, VISSA, UVE, and ICO were applied to select feature wavelengths from the SNV-pretreated spectra, and the results are shown in Figure 5 and Table S1. CARS retained 84 wavelengths, VISSA 67, UVE 54, and ICO 83. The exact calibrated wavelengths selected by each method are listed in Table S2.

CARS identified spectral regions mainly concentrated in the 400–500 nm and 850–1000 nm ranges, corresponding to myoglobin absorption bands and O-H overtone bands of water, respectively. These regions may contain information related to myoglobin absorption and O–H overtones of water [4,50]. VISSA selected wavelengths across both visible and near-infrared regions, with several bands located in the 650–850 nm region, which is associated with myofibrillar protein structure and textural properties. This region may reflect variation in myofibrillar protein structure and textural properties [9]. In contrast, UVE selected fewer wavelengths in the near-infrared region.

Among the intervals retained by ICO, two were located in the near-infrared region, 850–883 nm and 953–1003 nm. The first may contain information related to protein N–H stretching, while the second may reflect O–H absorption of water [4,50]. CQI integrates multiple quality dimensions, including meat color, water-holding capacity, and textural properties. The continuous intervals retained by ICO may therefore capture information from several of these attributes.

Although the four algorithms used different selection strategies, the retained wavelengths were mainly located in regions associated with meat color, water status, and protein structure. These results indicate that CQI-related spectral information was retained after variable selection. More than 60% of the original variables were removed, reducing spectral redundancy and simplifying subsequent modeling. Because CQI integrates multiple quality attributes, useful information may still remain outside the selected bands. The stability of these wavelengths was not tested by repeated resampling or in independent sample batches. Their application beyond the present dataset requires further validation. The effect of wavelength reduction on prediction performance was therefore examined further in the next section.

### 3.7. Establishment of the CQI Prediction Model

#### 3.7.1. Spectral-Level Model Comparison

To evaluate the prediction performance of different modeling methods for CQI, PLSR, BPNN, and XGBoost models were established using the SNV-preprocessed full spectra and selected characteristic wavelengths (Table 6). Among the three models, XGBoost achieved *R*_p_^2^ values above 0.80, outperforming PLSR and BPNN in the fixed spectral-level comparison. The XGBoost models also showed a marked calibration-prediction gap, with *R*_c_^2^ values near 0.998 and *R*_p_^2^ values around 0.87. The XGBoost parameters were tuned by randomized search with five-fold cross-validation, with L1 and L2 regularization also applied. The calibration-prediction gap still remained, and some overfitting may have been present. The spectral-level results were therefore considered together with the animal-level validation.

CQI brings together several quality attributes, including meat color, water-holding capacity, and texture. Color- and water-related information usually produces stronger spectral responses, whereas texture-related information is less prominent. The performance of XGBoost may be related to its ability to handle nonlinear relationships among variables with different signal strengths [40]. In contrast, PLSR tends to be dominated by strong-response variables [38], and may not fully exploit weaker texture-related signals. BPNN is able to capture nonlinear patterns, but with the limited sample size in this study, its performance may be more sensitive to overfitting and reduced generalization [39].

After feature wavelength selection, the prediction performance varied among model types. Most selected-wavelength PLSR models performed worse than the full-spectrum SNV-PLSR model, but ICO-PLSR gave a slight improvement. For BPNN and XGBoost, some selected-wavelength models, such as UVE-BPNN, VISSA-XGBoost, and ICO-XGBoost, performed comparably or slightly better. These results suggest that wavelength selection was beneficial only when the retained bands still contained enough CQI-related information. Because some quality-related signals, especially those linked to texture, are relatively weak in the spectra, different selection methods may not retain them to the same extent.

**Figure 6:**
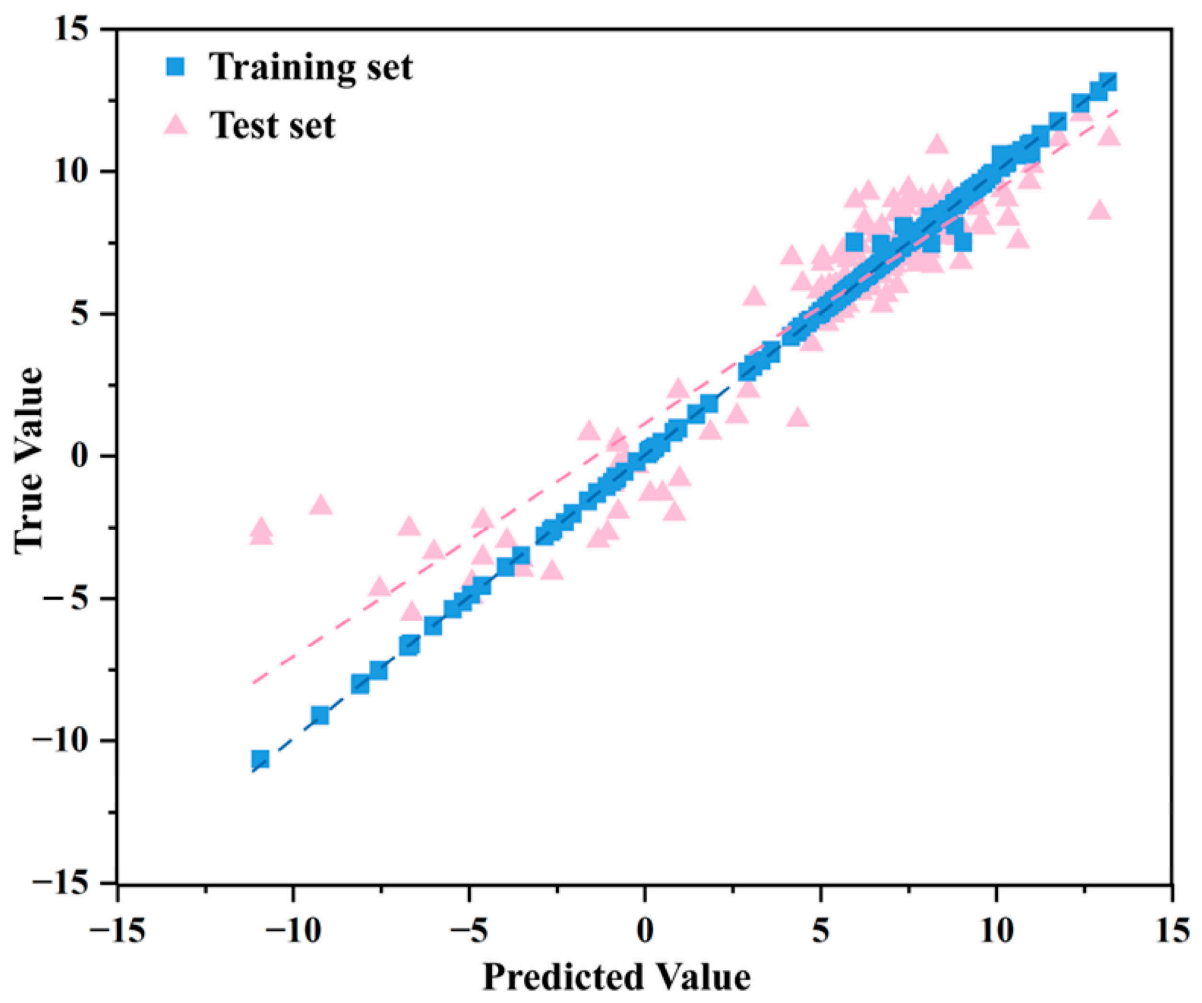
Calibration and prediction results of the SNV-ICO-XGBoost model for CQI prediction.

Among all evaluated models, SNV-ICO-XGBoost showed the numerically highest prediction performance (Figure 6), with an *R*_p_^2^ of 0.8670, an *RMSE*_P_ of 1.7319 and an *RPD*_p_ of 2.7423. Because *RPD*_p_ is calculated by dividing the standard deviation of the reference values by *RMSE*_P_, this value indicates that *RMSE*_P_ was approximately 36.5% of the CQI standard deviation in the prediction subset. The *RPD*_p_ was therefore considered together with *R*_p_^2^ and *RMSE*_P_ rather than interpreted using a fixed threshold alone.

SNV-VISSA-XGBoost gave a similar *R*_p_^2^ of 0.8652. The ICO-XGBoost model required 83 feature wavelengths, reducing the number of variables by 62.9%. The selected wavelengths were mainly located in spectral regions that may contain information associated with myoglobin, water, and protein, which matched the quality traits included in CQI. Considering its numerical performance and reduced number of variables, SNV-ICO-XGBoost was selected as the representative model for CQI prediction and visualization. The small difference between ICO-XGBoost and VISSA-XGBoost does not establish that ICO-XGBoost was clearly superior.

The same fixed prediction subset was used to compare preprocessing methods, wavelength-selection methods, and regression models. These spectral-level results therefore represent an internal model comparison rather than fully independent validation. Further testing with independent production batches is needed before practical application.

#### 3.7.2. Animal-Level Robustness Assessment

After the repeated spectra were averaged at the animal level, the models were evaluated again to check whether the CQI-related spectral information could still be captured. The results are summarized in Table 7.

PLSR gave the most stable prediction results, whereas XGBoost still showed acceptable predictive ability but with a wider gap between calibration and prediction performance. BPNN was more variable across the repeated partitions.

Averaging reduced part of the within-animal spectral variation, and each pig rather than each spectrum served as the modeling unit. This may partly explain the lower prediction performance. The averaged spectra still contained useful information for CQI prediction, with PLSR giving the highest and most stable mean animal-level performance. BPNN was more sensitive to repeated data partitioning.

All 224 wavelengths were retained in this assessment. ICO wavelength selection and full hyperparameter optimization were not repeated within each partition. The analysis therefore assessed the robustness of the full-spectrum animal-level models rather than repeatedly validating the complete SNV-ICO-XGBoost pipeline.

### 3.8. CQI Visualization

**Figure 7:**
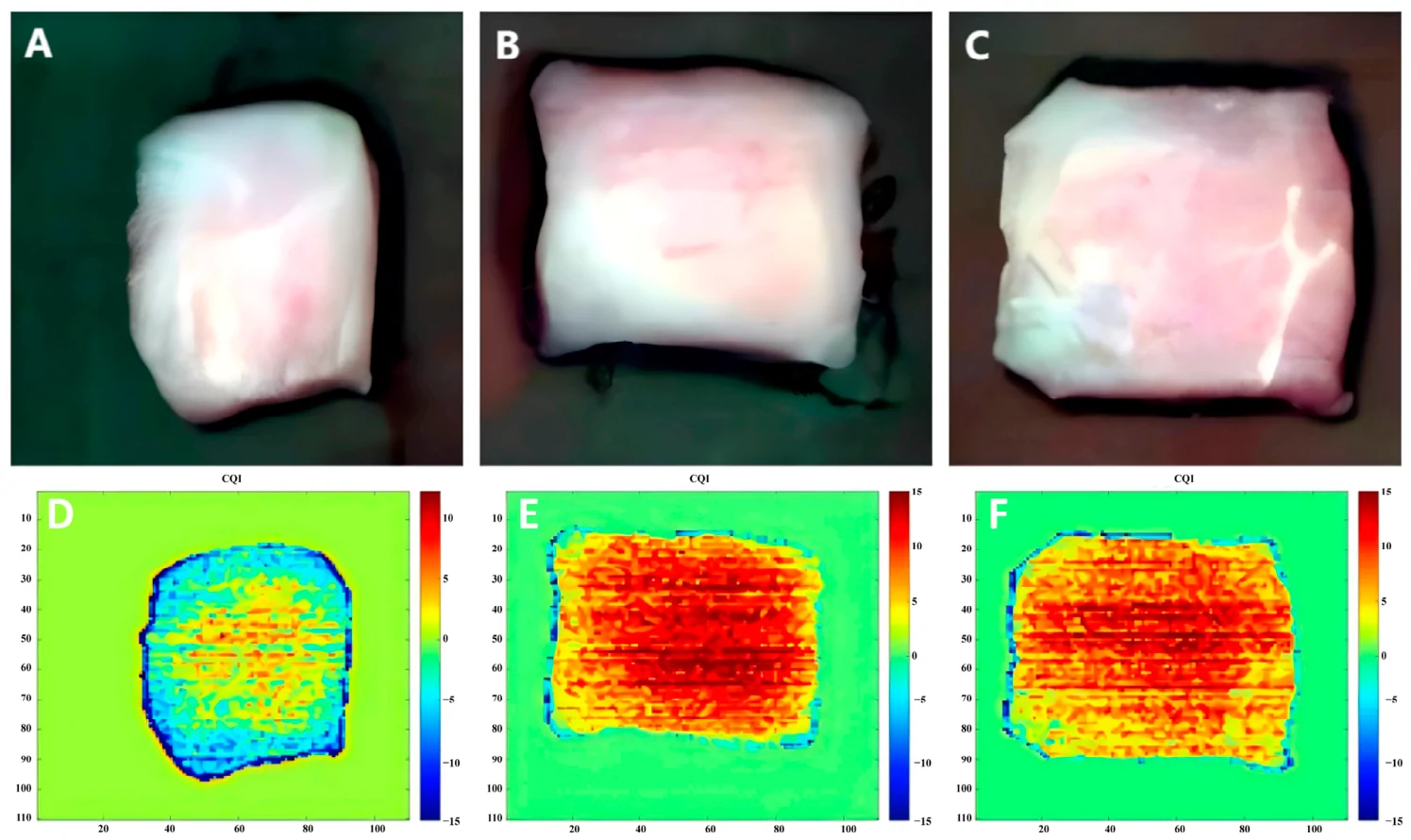
Pixel-wise maps of model-predicted CQI values in pork samples from Groups A, B, and C. Note: The samples from left to right are Group A (30–70 kg) (**A**,**D**), Group B (70–110 kg) (**B**,**E**), and Group C (110–150 kg) (**C**,**F**). The same color scale was used for all samples.

The SNV-ICO-XGBoost model was applied pixel-wise to generate CQI maps of the pork samples (Figure 7). A common color scale was used for all samples, with reddish-brown indicating higher predicted CQI values and deep blue indicating lower values. Groups B and C generally showed larger high-CQI regions than Group A, consistent with the sample-level results. Lower predicted CQI values were observed near the sample edges, possibly owing to surface curvature, illumination, or light-scattering effects. In the absence of pixel-level reference measurements, the maps were only used to illustrate spatial variation in model-predicted CQI values and were not interpreted as validated evidence of local quality heterogeneity.

## 4. Conclusions

This study developed a composite quality index (CQI) from instrumental traits related to color, water-holding capacity, texture, and antioxidant status in pigs from different body weight groups. The CQI represents a dataset-dependent summary of the selected instrumental traits and was not validated against sensory or consumer outcomes. The formulation of CQI also depends on the direction-based grouping of traits according to their PC1 loadings in the present dataset. Hyperspectral imaging combined with machine learning was then applied to predict CQI rapidly. In the fixed spectral-level comparison, SNV-ICO-XGBoost produced the numerically highest prediction performance, with an *R*_p_^2^ of 0.8670 and a *RPD*_p_ of 2.7423. Using averaged spectra, the repeated animal-level assessment showed that PLSR achieved the highest mean prediction performance, with an *R*_p_^2^ of 0.807 ± 0.058, an *RMSE*_p_ of 1.857 ± 0.345, and an *RPD*_p_ of 2.374 ± 0.345 across 50 random splits. These results provide a more conservative estimate of animal-level prediction performance than the fixed spectral-level comparison. Overall, the results indicate that variation in multiple instrumental pork-quality traits can be summarized by a composite index and predicted from hyperspectral data within the present dataset. The CQI maps qualitatively illustrate the spatial variation in model predictions. Further work should evaluate the CQI against independent sensory or consumer outcomes and validate the complete modeling pipeline using independent production batches, breeds, feeding systems, and postmortem conditions. Possible confounding by age, body weight, and production batch should also be considered.

## Figures and Tables

**Table 1:** Sample preparation scheme.

Test Items	Sample Specifications	Shape
L*, a* and b*	5 × 5 × 1 cm	Sliced parallel to the muscle fibers
pH	—	Direct electrode insertion technique
SF	1 × 1 × 2.5 cm	Cut into strips, following the direction of the muscle fibers
TPA	1.5 × 1 ×1 cm	Block
MC	2–10 g and less than 5 mm thick	Minced, sampled from the geometric center
WHC	2.5 cm diameter × 2 cm height	Cylindrical in shape, cut along the direction of the muscle fibers
DL	2.0 × 3.0 × 5.0 cm	strip-shaped
CL	6 × 4 × 4 cm	Block
T-AOC, T-SOD, MDA	About 3 g	Minced, sampled from the geometric center
HSI	4 × 4 × 1 cm	Sliced parallel to the muscle fibers

Note: For pH measurement, the electrode was inserted directly into a separate muscle piece, and no fixed sample dimensions were specified. SF and TPA samples should initially be vacuum-sealed in 6 × 4 × 4 cm blocks and heated in a water bath at 80 °C until the core temperature reaches 70 °C, which required approximately 20 min. After cooling to 0–4 °C, cut these samples to the dimensions specified in the table. For HSI samples, take three parallel samples from each pig. ‘—’ indicates not applicable.

**Table 2:** Instrumental pork-quality traits of different weight groups.

Trait	Group A (30–70 kg)	Group B (70–110 kg)	Group C (110–150 kg)
L*	49.375 ± 2.639 ^a^	53.060 ± 4.050 ^c^	51.300 ± 2.740 ^b^
a*	9.190 ± 1.210 ^a^	14.590 ± 1.650 ^c^	13.490 ± 1.860 ^b^
b*	3.880 ± 0.560 ^a^	5.930 ± 1.140 ^c^	4.430 ± 0.880 ^b^
pH	5.510 ± 0.110 ^c^	5.420 ± 0.110 ^b^	5.390 ± 0.090 ^a^
MC (%)	76.220 ± 0.190 ^c^	74.400 ± 0.220 ^b^	74.130 ± 0.230 ^a^
WHC (%)	76.770 ± 4.780 ^a^	85.850 ± 9.640 ^b^	86.250 ± 10.620 ^b^
DL	0.033 ± 0.010 ^b^	0.035 ± 0.011 ^c^	0.031 ± 0.009 ^a^
CL	0.214 ± 0.037 ^a^	0.222 ± 0.033 ^a^	0.216 ± 0.027 ^a^
SF (gf)	5568.310 ± 1111.080 ^a^	8078.260 ± 2498.420 ^b^	8157.600 ± 2527.150 ^b^
HD (gf)	4701.320 ± 1129.620 ^a^	7432.760 ± 1276.450 ^b^	7886.340 ± 1601.980 ^c^
SP	0.407 ± 0.062 ^a^	0.505 ± 0.042 ^b^	0.467 ± 0.045 ^ab^
CO	0.501 ± 0.099 ^c^	0.452 ± 0.032 ^a^	0.462 ± 0.031 ^ab^
GM (gf)	2366.2 ± 629.6 ^a^	3373.8 ± 688.3 ^b^	3632.0 ± 795.3 ^b^
CH (gf)	956.680 ± 275.220 ^a^	1702.400 ± 363.220 ^b^	1712.140 ± 433.310 ^b^
RE	0.148 ± 0.018 ^c^	0.132 ± 0.015 ^a^	0.137 ± 0.012 ^ab^
T-AOC (U/mg prot)	0.047 ± 0.012 ^b^	0.045 ± 0.010 ^ab^	0.042 ± 0.008 ^a^
T-SOD (U/mg prot)	14.570 ± 3.080 ^b^	12.240 ± 1.780 ^a^	12.330 ± 1.440 ^a^
MDA (nmol/mg prot)	0.116 ± 0.050 ^ab^	0.091 ± 0.023 ^b^	0.074 ± 0.022 ^a^

Note: Values are presented as mean ± SD (*n* = 76 animals per group). Within a row, different lowercase superscript letters indicate significant differences by Duncan’s multiple range test (*p* < 0.05). Values sharing at least one lowercase letter are not significantly different (*p* > 0.05). For cooking loss, one-way ANOVA showed no significant difference among the three groups (F_2,225_ = 1.240, *p* = 0.291).

**Table 3:** Total Variance Explained.

Component	Initial Eigenvalues			Extraction SSL			Rotation SSL		
	Total	Var.%	Cum.%	Total	Var.%	Cum.%	Total	Var.%	Cum.%
1	4.738	36.445	36.445	4.738	36.445	36.445	3.353	25.790	25.790
2	2.177	16.743	53.188	2.177	16.743	53.188	2.054	15.803	41.592
3	1.428	10.983	64.170	1.428	10.983	64.170	2.019	15.528	57.120
4	1.229	9.452	73.622	1.229	9.452	73.622	1.656	12.737	69.857
5	1.047	8.056	81.678	1.047	8.056	81.678	1.537	11.821	81.678
6	0.669	5.143	86.820						
7	0.527	4.050	90.871						
8	0.359	2.765	93.636						
9	0.306	2.357	95.994						
10	0.249	1.915	97.909						
11	0.207	1.590	99.499						
12	0.059	0.452	99.951						
13	0.006	0.049	100.00						

Abbreviations: SSL, Sums of Squared Loadings; Var.%, percentage of variance; Cum.%, cumulative percentage.

**Table 4:** CQI and CWI values of pork from different body weight groups.

Indicator	Group A (30–70 kg)	Group B (70–110 kg)	Group C (110–150 kg)
CQI	−2.564 ± 3.504 ^a^	7.658 ± 2.120 ^b^	7.051 ± 2.087 ^b^
CWI	0.284 ± 0.074 ^a^	0.410 ± 0.067 ^b^	0.414 ± 0.073 ^b^

Note: Different lowercase letters within the same row indicate significant differences between groups (*p* < 0.05); values sharing at least one lowercase letter are not significantly different (*p* > 0.05).

**Table 5:** CQI prediction by PLSR with different preprocessing methods.

Model	Band	LV_s_	*RMSE*_c_	*R*_c_^2^	*RMSE*_p_	*R_p_^2^*	*RPD*_p_
O-PLSR	397.66–1003.81 nm	27	1.7238	0.8414	2.0187	0.8194	2.3528
SG-PLSR	397.66–1003.81 nm	24	1.7427	0.8379	2.0754	0.8091	2.2885
SNV-PLSR	397.66–1003.81 nm	20	1.5908	0.8649	1.8795	0.8434	2.5271
MSC-PLSR	397.66–1003.81 nm	25	1.4258	0.8825	2.0803	0.8393	2.4944
Base-PLSR	397.66–1003.81 nm	23	1.5846	0.8549	2.2770	0.8075	2.2789

Note: O, original spectra; Base, baseline correction; LV_s_, latent variables. *RPD* was calculated as the population standard deviation of the reference CQI values in the prediction subset divided by *RMSE*_P_, with n used as the denominator.

**Table 6:** CQI prediction by different models with feature wavelength subsets.

Model	Variable Number	*RMSE*_c_	*R*_c_^2^	*RMSE*_p_	*R_p_^2^*	*RPD*_p_
SNV-PLSR	224	1.5908	0.8649	1.8795	0.8434	2.5271
CARS-PLSR	84	1.7668	0.8334	2.1996	0.7855	2.1592
VISSA-PLSR	67	1.7309	0.8401	2.0740	0.8093	2.2900
UVE-PLSR	54	1.8589	0.8156	2.1606	0.7931	2.1983
ICO-PLSR	83	1.4655	0.8854	1.7901	0.8580	2.6533
SNV-BPNN	224	2.0356	0.7976	1.9633	0.7810	2.1369
CARS-BPNN	84	1.8735	0.8285	1.7407	0.8278	2.4101
VISSA-BPNN	67	1.7861	0.8442	1.8055	0.8148	2.3236
UVE-BPNN	54	1.6729	0.8633	1.5675	0.8604	2.6764
ICO-BPNN	83	1.7530	0.8499	1.7950	0.8169	2.3372
SNV-XGBoost	224	0.1795	0.9981	2.0199	0.8485	2.5690
CARS-XGBoost	84	0.1821	0.9978	2.1499	0.8283	2.4136
VISSA-XGBoost	67	0.1665	0.9985	1.7439	0.8652	2.7235
UVE-XGBoost	54	0.1672	0.9983	1.8492	0.8484	2.5648
ICO-XGBoost	83	0.1663	0.9985	1.7319	0.8670	2.7423

Note: *RPD* was calculated as the population standard deviation of the reference CQI values in the prediction subset divided by *RMSE*_p_, with n used as the denominator.

**Table 7:** Animal-level robustness assessment of full-spectrum models for CQI prediction.

Model	*R*_c_^2^	*RMSE*_c_	*R*_p_^2^	*RMSE*_p_	*RPD*_p_
PLSR	0.866 ± 0.015	1.624 ± 0.129	0.807 ± 0.058	1.857 ± 0.345	2.374 ± 0.345
BPNN	0.492 ± 0.403	2.898 ± 1.222	0.269 ± 0.412	3.519 ± 0.843	1.288 ± 0.292
XGBoost	0.914 ± 0.012	1.300 ± 0.101	0.749 ± 0.075	2.117 ± 0.384	2.083 ± 0.316

## Data Availability

The data presented in this study are not publicly available due to restrictions associated with the research project and collaborating parties. The data may be available from the corresponding authors upon reasonable request and with permission from the relevant parties.

## Supplementary Materials

The following supporting information can be downloaded at: https://www.mdpi.com/article/10.3390/foods15162892/s1, Method S1: Determination of Myofibrillar Proteins; Method S2: Detailed Parameters for Hyperspectral Data Acquisition; Method S3: Detailed CQI Calculation Procedure; Method S4: Spectral Preprocessing Procedures; Figure S1: Secondary structure composition of myofibrillar proteins from pork longissimus dorsi in different groups; Figure S2: Intrinsic fluorescence spectra of myofibrillar proteins from pork longissimus dorsi in different groups (excitation wavelength 280 nm; Figure S3: Scree plot of the principal component analysis based on the 13 retained instrumental pork-quality traits. The dashed horizontal line indicates an eigenvalue of 1; Table S1: Implementation settings of the wavelength-selection algorithms; Table S2: Exact calibrated wavelengths retained by the four wavelength-selection methods; Table S3: Implementation settings of the spectral-level PLSR and BPNN models; Table S4: XGBoost hyperparameter search space and final model settings; Table S5: Data preparation and model settings for the repeated animal-level assessment; Table S6: Myofibrillar fragmentation index (MFI) and chemical force measurements of pork longissimus dorsi from different groups; Table S7: Secondary structure content of myofibrillar proteins from pork longissimus dorsi in different groups; Table S8: Results of the KMO and Bartlett’s sphericity test for principal component analysis; Table S9: Variable communalities; Table S10: Rotated component matrix of pork quality indicators; Table S11: Component score coefficients, overall scores, and normalized scoring coefficients of quality indicators.

## Abbreviations

The following abbreviations are used in this manuscript:

HSI	Hyperspectral imaging
CQI	Composite quality index
CWI	Comprehensive weight index
ICO	Interval combination optimization
XGBoost	Extreme gradient boosting
PLSR	Partial least squares regression
BPNN	Backpropagation neural network
SNV	Standard normal variate
SG	Savitzky–Golay
MSC	Multiplicative scatter correction
CARS	Competitive adaptive reweighted sampling
VISSA	Variable iterative space shrinkage approach
UVE	Uninformative variable elimination
SPXY	Sample set partitioning based on joint X-Y distances
NIR	Near-infrared
WHC	Water-holding capacity
DL	Drip loss
CL	Cooking loss
MC	Moisture content
SF	Shear force
TPA	Texture profile analysis
HD	Hardness
SP	Springiness
CO	Cohesiveness
GM	Gumminess
CH	Chewiness
RE	Resilience
T-AOC	Total antioxidant capacity
T-SOD	Total superoxide dismutase
MDA	Malondialdehyde
MFI	Myofibrillar fragmentation index
*R*^2^	Coefficient of determination
*RMSE*	Root mean square error
*RPD*	relative percentage deviation
