# Supplementary material for: Development of a Composite Pork Quality Index from Instrumental Traits and Its Hyperspectral Prediction
Source: Foods. 2026 Aug 18;15(16):2892. doi: 10.3390/foods15162892 (PMC13511944; doi:10.3390/foods15162892)
Supplement: Supplementary file 1 [file foods-15-02892-s001.zip › foods-4461356-supplementary.pdf]

Development of a composite pork quality index from instrumental traits and its hyperspectral prediction

Yiqi Rao [1,†], Yongzhe He [2,†], Siyao Huang[3], Qian You[2], Shuqi Tang[4], Hu Zhang[4], Liandong Luo[1], Xiaoyan Xu[2,*], Xingguo Tian[1,*]

[1] The National Center for Precision Machining and Safety of Livestock and Poultry Products Joint Engineering Research Center, College of Food Science, South China Agricultural University, Guangzhou 510642, China
[2] Guangdong Provincial Key Laboratory of Food Quality and Safety, College of Food Science, South China Agricultural University, Guangzhou 510642, China
[3] College of Mathematics and Informatics, South China Agricultural University, Guangzhou 510642, China
[4] College of Engineering, South China Agricultural University, Guangzhou 510642, China

Correspondence: yanzixu2001@scau.edu.cn (X.X.); xingguot@scau.edu.cn (X.T.)
[†] These authors contributed equally to this work.

Method S1. Determination of Myofibrillar Proteins

Extraction of Myofibrillar Proteins

Six pigs were randomly selected from each body weight group for myofibrillar protein extraction following [1]. Minced meat (4 g) was mixed with 20 mL of phosphate buffer I (1 mmol/L EGTA, 2 mmol/L $MgCl_2$, 100 mmol/L NaCl, 10 mmol/L K_3PO_4, pH 7.0) and homogenized using a high-speed dispersing homogenizer (FJ200-SH, Shanghai Specimen Model Factory, China) in an ice bath at 7,000 rpm for 1 min. The homogenate was centrifuged at 6,000 rpm for 15 min at 4 °C, and the supernatant was discarded. The precipitate was resuspended in 20 mL of phosphate buffer I, and the homogenization and centrifugation steps were repeated three times. After the final centrifugation, the precipitate was collected, mixed with 20 mL of 0.1 mol/L NaCl solution (pH 6.0), and homogenized at 7,000 rpm for 30 s. The homogenate was filtered through two layers of 100-mesh gauze, and the filtrate was centrifuged at 6,000 rpm for 15 min at 4 °C. The precipitate was resuspended in 0.1 mol/L NaCl solution and washed three times. After discarding the supernatant, the final precipitate was collected as myofibrillar proteins. All operations were performed under ice-bath conditions.

Determination of Myofibrillar Fragmentation Index (MFI)

MFI was determined as described in [1]. The myofibrillar protein precipitate was dissolved in phosphate buffer II (0.02 mol/L PBS, 0.6 mol/L NaCl, pH 6.0), and the protein concentration was adjusted to 0.5 mg/mL using the biuret method. The absorbance was measured at 540 nm using a UV-1800 UV-Vis spectrophotometer (UV-1800, Shimadzu, Japan). The average of three absorbance readings was multiplied by 200 to obtain the MFI (MFI = $A_{540} \times 200$).

Circular Dichroism (CD) Spectroscopy

The myofibrillar protein precipitate was dissolved in phosphate buffer II (0.02 mol/L PBS, 0.6 mol/L NaCl, pH 6.0), and the protein concentration was adjusted to 0.5 mg/mL using the biuret method. CD spectra were recorded using a Chirascan-plus V100 circular dichroism spectrometer (Chirascan-plus V100, Applied Photophysics, UK) over the range of 190-240 nm with a step size of 1.0 nm. The CD data were analyzed using CDNN software to calculate the relative contents of α-helix, β-sheet, β-turn, and random coil.

Intrinsic Fluorescence Spectroscopy

The myofibrillar protein precipitate was dissolved in phosphate buffer II (0.02 mol/L PBS, 0.6 mol/L NaCl, pH 6.0), and the protein concentration was adjusted to 0.5 mg/mL using the biuret method. Intrinsic fluorescence spectra

were recorded using an F-7100 fluorescence spectrophotometer (F-7100, Hitachi, Japan) over the emission wavelength range of 250-500 nm with an excitation wavelength of 280 nm, sensitivity of 5, and slit width of 5 nm.

Determination of Chemical Forces

Chemical forces were determined as described in [2]. The myofibrillar protein precipitate was dissolved in phosphate buffer II (0.02 mol/L PBS, 0.6 mol/L NaCl, pH 6.0), and the protein concentration was adjusted to 5 mg/mL using the biuret method. The following solutions were prepared: 0.05 mol/L NaCl (SA), 0.6 mol/L NaCl (SB), 0.6 mol/L NaCl + 1.5 mol/L urea (SC), 0.6 mol/L NaCl + 8 mol/L urea (SD), and 0.6 mol/L NaCl + 8 mol/L urea + 0.05 mol/L β-mercaptoethanol (SE). Aliquots of 2 mL of the myofibrillar protein solution were mixed with 10 mL of each of the five solutions, respectively, and allowed to stand at 4 °C for 1 h. The mixtures were then centrifuged at 10,000 rpm for 15 min at 4 °C. The protein concentration in each supernatant was determined by the biuret method. The protein solubility in the SA solution represents the contribution of van der Waals forces. The difference in solubility between SA and SB represents the contribution of ionic bonds, between SB and SC represents hydrogen bonds, between SC and SD represents hydrophobic interactions, and between SD and SE represents disulfide bonds.

Method S2. Detailed Parameters for Hyperspectral Data Acquisition

Hyperspectral images were acquired using a line-scanning system consisting of an FX10 hyperspectral camera (SPECIM, Spectral Imaging Ltd., Oulu, Finland), a 280 W halogen light source (DECOSTAR 51S, Osram Corp., Germany), a motorized stage (HXY-OFX01, Red Star Yang Technology Corp., Wuhan, China), and two computers: one for controlling the stage movement and the other equipped with Lumo-Scanner software (SPECIM, Spectral Imaging Ltd., Oulu, Finland) for controlling the acquisition parameters. The hyperspectral camera operated in line-scanning mode, covering a spectral range of 400-1000 nm with 224 wavebands and a spectral resolution of 2.7 nm. The acquisition parameters were as follows: stage movement speed of 9.8 mm/s, CCD camera exposure time of 2 ms, and a distance of 32 cm between the lens and the sample surface. The overall layout of the system is shown in Fig. 1 of the main text.

The acquired hyperspectral images were corrected to reflectance mode using black-and-white correction [3]. The white reference image was obtained by collecting a uniform, stable, high-reflectance Teflon tile, and the dark reference image was obtained by turning off the light source and covering the lens. The correction formula is as follows:

$$R=\frac{I_0 - I_B}{I_W - I_B}\times 100\% \tag{S1}$$

Where R represents the calibrated hyperspectral image, I_O indicates the raw hyperspectral image, I_B denotes the black reference image, and I_W stands for the white reference image.

The corrected images were processed using ENVI 4.8 software (ITT Visual Information Solutions, Boulder, CO, USA). A threshold masking method was used to separate the sample from the background: the difference image between bands 212 and 93 was calculated, and a segmentation threshold of 0.03 was applied to create a binary mask for the region of interest (ROI). The average spectral reflectance within the ROI was then extracted. Three replicate HSI samples were prepared per pig, each measuring 4 × 4 × 1 cm and cut along the muscle fiber direction.

Method S3. Detailed CQI Calculation Procedure

The CQI inputs were animal-level instrumental trait values rather than z-scores. Replicate measurements were first averaged within each pig. Each retained trait was then min–max normalized across all 228 pigs according to Equation (5) in the main manuscript. An offset of 0.001 was added to each normalized value to ensure that all inputs remained strictly positive for subsequent multiplication, division, and natural-logarithmic transformation. No absolute-value transformation was applied, and the dataset contained no missing values.

One CQI value was calculated separately for each pig. The traits with positive PC1 loadings (L*, a*, b*, WHC, SF, HD, GM, and CH) entered the numerator, whereas those with negative PC1 loadings (T-AOC, T-SOD, MC, CO, and RE) entered the denominator. The loading signs were used only as a mathematical grouping rule and were not interpreted as favorable or unfavorable biological directions.

Each of the 13 retained traits entered the CQI expression once, corresponding to an exponent of one. Loading magnitudes were not used as weights because no independent sensory or biological evidence was available to justify unequal exponents. This mathematical treatment does not imply that all retained traits have equal biological importance.

The calculation procedure can be expressed as follows:

For each pig i and retained trait j:

$$u[i,j] = 0.001 + (x[i,j] - \min[xj]) / (\max[xj] - \min[xj])$$

$$\text{numerator}[i] = u[i,L^*] \times u[i,a^*] \times u[i,b^*] \times u[i,WHC] \times u[i,SF] \times u[i,HD] \times u[i,GM] \times u[i,CH]$$

$$\text{denominator}[i] = u[i,T\text{-}AOC] \times u[i,T\text{-}SOD] \times u[i,MC] \times u[i,CO] \times u[i,RE]$$

$$CQI[i] = 1000 \times \ln(\text{numerator}[i] / \text{denominator}[i])$$

The constant factor 1000 was applied identically to all pigs. It rescales the CQI values to a more convenient numeric range without altering pig ranking, pairwise differences in log-ratio, or correlations with any external variable.

Method S4. Spectral Preprocessing Procedures

Four preprocessing approaches were compared using the calibrated reflectance spectra: standard normal variate transformation (SNV), Savitzky–Golay smoothing (SG), multiplicative scatter correction (MSC), and linear baseline correction. The calibrated spectra contained 224 wavelengths spanning 397.66–1003.81 nm. No additional terminal-band removal was performed. Each preprocessing approach was applied separately, and the same fixed calibration and prediction subsets were used for model comparison.

SNV was applied independently to each spectrum by subtracting its mean reflectance and dividing by its standard deviation across the 224 retained wavelengths.

SG smoothing was performed using an 11-point window and a second-order polynomial. The derivative order was set to zero; thus, SG was used only for smoothing and not for spectral differentiation. The default end-point treatment in The Unscrambler X 10.4 was used.

For MSC, the mean spectrum of the calibration subset was used as the reference spectrum. Each calibration and prediction spectrum was regressed against this reference, and the fitted additive and multiplicative terms were used for correction. The prediction spectra were corrected using the calibration-derived reference spectrum.

Linear baseline correction was performed separately for each spectrum. A straight baseline connecting the reflectance values at the first and last retained wavelengths was estimated and subtracted from the spectrum.

The preprocessing comparison was conducted using PLSR. The number of latent variables was selected within the calibration subset by five-fold cross-validation according to the minimum $RMSE_{CV}$.

Supplementary Figures and Tables

Table S1. Implementation settings of the wavelength-selection algorithms.

Method	Retained variables	Iteration and resampling	Validation	Selection or stopping rule	Seed
CARS	84	50 Monte Carlo runs; 80% of calibration samples; exponentially decreasing function and adaptive reweighted sampling	Five-fold PLSR cross-validation	Minimum RMSECV	42
VISSA	67	39 iterations; 500 weighted binary submodels per iteration; 80% sample proportion	Five-fold PLSR cross-validation	Minimum RMSECV; stopped after five consecutive non-improving iterations	42
UVE	54	224 artificial noise variables; 100 repeated noise generations	Leave-one-out validation	Variable stability greater than the maximum absolute stability of noise variables	42
ICO	83	40 initial intervals; interval width of approximately 5–6 bands; 100 submodel evaluations; maximum 50 iterations	Five-fold PLSR cross-validation	Minimum RMSECV	42

Abbreviations: CARS, competitive adaptive reweighted sampling; VISSA, variable iterative space shrinkage approach; UVE, uninformative variable elimination; ICO, interval combination optimization; PLSR, partial least squares regression; RMSECV, root mean square error of cross-validation.

Table S2. Exact calibrated wavelengths retained by the four wavelength-selection methods.

Method	Number of retained wavelengths	Retained calibrated wavelengths (nm)
CARS	84	397.66; 408.13–418.62; 423.86–431.74; 437.00; 447.52; 463.34–473.90; 484.47–487.12; 540.24; 569.61; 577.64–583.00; 588.36; 604.47; 609.85; 615.23; 634.08–636.78; 642.18–644.88; 650.29; 655.69–663.81; 682.79–685.50; 690.93; 699.09; 707.25–709.97; 715.42; 720.87–729.06; 734.52–748.19; 861.14; 880.58; 886.15–916.81; 925.19–930.78; 936.38–941.98; 950.38–953.19; 964.41–972.84; 984.09; 995.35; 1000.99–1003.81.
VISSA	67	416.00–418.62; 426.49; 434.37; 439.63; 447.52; 458.06; 473.90; 479.18; 492.42; 510.98; 518.95; 529.59; 534.91; 540.24; 545.57–550.91; 556.25; 580.32; 604.47; 609.85; 615.23–617.92; 636.78; 666.52; 677.36–688.22; 709.97; 718.15; 723.60; 737.25–739.98; 748.19–753.66; 772.85; 778.34; 783.84–789.33; 814.12; 819.64; 825.16–833.45; 847.29–852.83; 875.03; 886.15–888.93; 939.18; 955.99; 961.60; 972.84–978.46; 984.09–989.72; 1000.99–1003.81.
UVE	54	447.52; 455.43–463.34; 492.42; 521.61–548.24; 577.64–593.73; 604.47–612.53; 628.69–652.99; 690.93–696.37; 701.81; 745.45–753.66; 786.58–794.84; 850.06–852.83; 886.15; 975.65.
ICO	83	397.66–426.49; 503.02–580.32; 726.33–748.19; 850.06–883.37; 953.19–1003.81.

Abbreviations: CARS, competitive adaptive reweighted sampling; VISSA, variable iterative space shrinkage approach; UVE, uninformative variable elimination; ICO, interval combination optimization.

Table S3. Implementation settings of the spectral-level PLSR and BPNN models.

Model	Parameter	Setting
PLSR	Candidate latent variables	1–20
PLSR	Validation	Five-fold cross-validation within the calibration subset
PLSR	Selection criterion	Minimum RMSECV
SNV-PLSR	Final latent variables	20
CARS-PLSR	Final latent variables	18
VISSA-PLSR	Final latent variables	19
UVE-PLSR	Final latent variables	17
ICO-PLSR	Final latent variables	15
BPNN	Hidden layers	64 and 32 neurons
BPNN	Activation	Hyperbolic tangent
BPNN	Optimizer	Adam
BPNN	Initial learning rate	0.001
BPNN	L2 regularization	0.0001
BPNN	Maximum iterations	2000
BPNN	Internal validation fraction	0.10
BPNN	Early-stopping patience	20 iterations
BPNN	Random seed	42
BPNN	Initialization	One fixed initialization

Abbreviations: PLSR, partial least squares regression; BPNN, backpropagation neural network; SNV, standard normal variate; CARS, competitive adaptive reweighted sampling; VISSA, variable iterative space shrinkage approach; UVE, uninformative variable elimination; ICO, interval combination optimization; $RMSE_{CV}$, root mean square error of cross-validation.

Table S4. XGBoost hyperparameter search space and final model settings.

Parameter					
n estimators	150, 200, 250, 300, 400, 500, 700	150	400	500	250
learning rate	0.01, 0.02, 0.03, 0.05, 0.07, 0.10	0.07	0.02	0.03	0.03
max depth	2, 3, 4, 5, 6, 7	6	6	6	6
min child weight	1, 2, 3, 5, 8, 12, 20	5	5	8	3
subsample	0.60, 0.70, 0.80, 0.90, 1.00	0.6	0.6	0.7	0.8
colsample bytree	0.60, 0.70, 0.80, 0.90, 1.00	0.8	0.9	0.6	0.8
gamma	0, 0.05, 0.10, 0.20, 0.50, 1.00, 2.00	0.2	0.05	0.5	2
reg alpha	0, 0.001, 0.01, 0.10, 0.50, 1.00	0	0.01	0.5	0
reg lambda	0.50, 1.00, 2.00, 5.00, 10.00	0.5	0.5	10	1

Note: Hyperparameters were optimized separately for each wavelength subset using randomized search with five-fold cross-validation. Fifty parameter combinations were evaluated, and the combination yielding the lowest cross-validated RMSE was retained. The squared-error objective and a random seed of 42 were used. **Abbreviations:** XGBoost, extreme gradient boosting; CARS, competitive adaptive reweighted sampling; VISSA, variable iterative space shrinkage approach; UVE, uninformative variable elimination; ICO, interval combination optimization.

Table S5. Data preparation and model settings for the repeated animal-level assessment.

Item	Setting
Biological unit	Individual animal
Spectral input	Mean of the three replicate spectra from each animal
Number of input variables	All 224 calibrated bands
Partitioning	50 repeated random 75%/25% animal-level splits
Random seeds	0–49
PLSR preprocessing	Standardization of spectral predictors
PLSR latent variables	1–20, selected within each training partition by five-fold cross-validation
PLSR selection criterion	Minimum cross-validated RMSE
BPNN preprocessing	Standardization of spectral predictors
BPNN hidden layers	32 and 16 neurons
BPNN activation	ReLU
BPNN optimizer	Adam
BPNN L2 coefficient	0.001
BPNN learning rate	0.001
BPNN maximum iterations	3000
BPNN early stopping	Applied
XGBoost n estimators	200
XGBoost learning rate	0.03
XGBoost max depth	2
XGBoost min child weight	12
XGBoost subsample	0.70
XGBoost colsample bytree	0.50
XGBoost gamma	0.50
XGBoost reg alpha	0.50
XGBoost reg lambda	2.00
Wavelength selection	ICO was not repeated
Hyperparameter optimization	Complete optimization was not repeated
RPD calculation	Population SD of prediction-reference CQI divided by RMSEP

Note: The repeated animal-level evaluation retained all 224 spectral bands and did not repeat ICO wavelength selection or the complete hyperparameter-optimization procedure within each partition. Therefore, it evaluated full-spectrum animal-level models rather than independently validating the complete SNV–ICO–XGBoost pipeline. **Abbreviations:** PLSR, partial least squares regression; BPNN, backpropagation neural network; XGBoost, extreme gradient boosting; ICO, interval combination optimization; CQI, composite quality index; $RMSE_P$, root mean square error of prediction; *RPD*, relative percentage deviation.

Table S6. Myofibrillar fragmentation index (MFI) and chemical force measurements of pork *longissimus dorsi* from different groups.

Parameter	Group A (30-70 kg)	Group B (70-110 kg)	Group C (110-150 kg)
MFI	50.8700 ± 4.9300^{c}	29.1600 ± 2.9900^{b}	22.2300 ± 1.5300^{a}
Van der Waals forces (mg/mL)	0.0709 ± 0.0201^{b}	0.0050 ± 0.0010^{a}	0.0184 ± 0.0040^{ab}
Ionic bonds (mg/mL)	0.2199 ± 0.0362^{a}	0.6702 ± 0.1236^{b}	0.6624 ± 0.0283^{b}
Hydrogen bonds (mg/mL)	0.1631 ± 0.0100^{a}	0.4043 ± 0.0460^{b}	0.5248 ± 0.0892^{c}
Hydrophobic interactions (mg/mL)	0.9433 ± 0.1740^{b}	0.5035 ± 0.0362^{a}	0.5745 ± 0.0967^{a}
Disulfide bonds (mg/mL)	0.4894 ± 0.1253^{a}	0.8936 ± 0.2135^{b}	1.1986 ± 0.1841^{c}

Note: Different lowercase letters within the same row indicate significant differences among groups ($p < 0.05$). MFI = $A_{540} \times 200$; values sharing at least one lowercase letter are not significantly different ($p > 0.05$).

Table S7. Secondary structure content of myofibrillar proteins from pork *longissimus dorsi* in different groups.

Parameter	Group A (30-70 kg)	Group B (70-110 kg)	Group C (110-150 kg)
α-helix (%)	20.97 ± 1.99^{a}	21.22 ± 2.29^{a}	25.48 ± 2.13^{b}
β-sheet (%)	13.90 ± 3.21^{a}	24.60 ± 3.51^{b}	16.85 ± 2.62^{a}
β-turn (%)	26.38 ± 0.75^{b}	25.20 ± 0.47^{a}	24.65 ± 0.18^{a}
Random coil (%)	37.17 ± 2.55^{c}	29.28 ± 0.65^{a}	32.70 ± 1.49^{b}

Note: Different lowercase letters within the same row indicate significant differences among groups ($p < 0.05$); values sharing at least one lowercase letter are not significantly different ($p > 0.05$).

Table S8. Results of the KMO and Bartlett's sphericity test for principal component analysis

KMO Sampling Adequacy Measure		0.706
Bartlett's Test of Sphericity	Approximate chi-square	2532.697
	Degrees of freedom	78
	Significance	<0.001

Table S9. Variable communalities

Physical and Chemical Properties	Initial	Extraction
*L**	1	0.811
*a**	1	0.752
*b**	1	0.840
T-AOC	1	0.776
T-SOD	1	0.728
MC	1	0.858
WHC	1	0.814
SF	1	0.567
HD	1	0.925
CO	1	0.860
GM	1	0.850
CH	1	0.922
RE	1	0.814

Abbreviations: *L**, *a**, *b**, CIE lightness, redness, and yellowness; T-AOC, total antioxidant capacity; T-SOD, total superoxide dismutase; MC, moisture content; WHC, water holding capacity; SF, shear force; HD, hardness; CO, cohesiveness; GM, gumminess; CH, chewiness; RE, resilience.

Table S10. Rotated component matrix of pork quality indicators

Physical and Chemical Properties	PC1	PC2	PC3	PC4	PC5
L^*	0.420	-0.433	0.403	-0.517	-0.134
a^*	0.783	-0.308	0.207	0.028	0.033
b^*	0.564	-0.450	0.445	-0.344	-0.048
T-AOC	-0.242	-0.249	0.630	0.496	0.114
T-SOD	-0.483	0.051	0.604	0.351	0.062
MC	-0.502	-0.011	0.122	-0.273	0.719
WHC	0.696	-0.099	-0.025	0.361	-0.435
SF	0.545	0.206	0.014	0.415	0.234
HD	0.860	0.359	-0.003	0.009	0.237
CO	-0.417	0.662	0.290	-0.274	-0.298
GM	0.764	0.572	0.087	-0.080	0.161
CH	0.830	0.438	0.141	-0.040	0.140
RE	-0.345	0.699	0.369	-0.108	-0.241

Abbreviations: L^*, a^*, b^*, CIE lightness, redness, and yellowness; T-AOC, total antioxidant capacity; T-SOD, total superoxide dismutase; MC, moisture content; WHC, water holding capacity; SF, shear force; HD, hardness; CO, cohesiveness; GM, gumminess; CH, chewiness; RE, resilience. PC, Principal component.

Table S11. Component score coefficients, overall scores, and normalized scoring coefficients of quality indicators

Physical and Chemical Properties	PC 1 25.790%	PC 2 15.803%	PC3 15.528%	PC4 12.737%	PC5 11.821%	Overall Score	Normalized scoring coefficient (%)
L^*	-0.113	0.534	0.129	-0.052	-0.061	0.075	7.36%
a^*	0.082	0.192	-0.113	0.044	0.106	0.064	6.24%
b^*	-0.038	0.475	0.047	-0.049	0.052	0.089	8.68%
T-AOC	0.063	0.026	-0.069	0.032	0.612	0.105	10.31%
T-SOD	0.059	0.006	0.095	-0.003	0.512	0.111	10.91%
MC	0.163	0.046	-0.140	-0.699	0.049	-0.068	-6.67%
WHC	-0.035	-0.044	-0.013	0.522	0.081	0.071	6.96%
SF	0.271	-0.211	-0.162	0.001	0.205	0.044	4.28%
HD	0.307	-0.035	-0.031	-0.117	-0.30	0.023	2.21%
CO	-0.021	0.098	0.505	0.069	-0.050	0.112	10.95%
GM	0.314	-0.003	0.120	-0.108	0.047	0.111	10.89%
CH	0.294	0.035	0.085	-0.072	0.002	0.105	10.26%
RE	0.048	0.044	0.469	0.083	0.075	0.137	13.37%

Note: The overall score was obtained by combining the component score coefficients with the variance contribution of each retained component. The last column is the normalized scoring coefficient used for CWI calculation. Negative values reflect the direction of contribution in the weighted summation. In the CQI calculation, positive and negative contributor groups were defined according to the loading signs on PC1 (Table S10). **Abbreviations:** L^*, a^*, b^*, CIE lightness, redness, and yellowness; T-AOC, total antioxidant capacity; T-SOD, total superoxide dismutase; MC, moisture content; WHC, water holding capacity; SF, shear force; HD, hardness; CO, cohesiveness; GM, gumminess; CH, chewiness; RE, resilience. PC, Principal component.

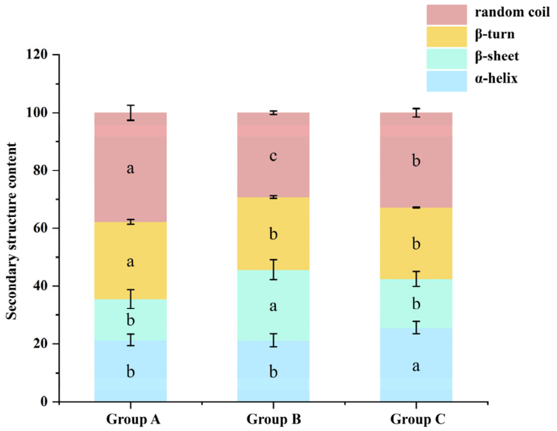

Figure S1. Secondary structure composition of myofibrillar proteins from pork longissimus dorsi in different groups.

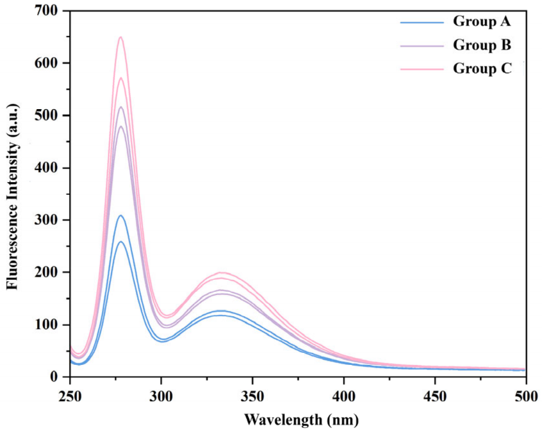

Figure S2. Intrinsic fluorescence spectra of myofibrillar proteins from pork longissimus dorsi in different groups (excitation wavelength 280 nm).

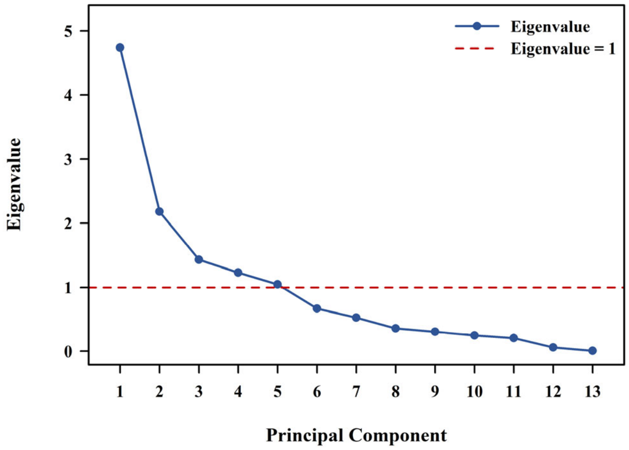

Figure S3. Scree plot of the principal component analysis based on the 13 retained instrumental pork-quality traits. The dashed horizontal line indicates an eigenvalue of 1.

References

1. Zhang, X.; Zhu, L.; Song, L.; Song, L.; Shi, S.; Liu, H.; Wu, J.; Si, K.; Gong, T.; Liu, H. Combined treatment of lactic acid-ultrasound-papain on yak meat and its tenderization mechanism. *Meat Science* **2023**, *196*, 109043, doi:10.1016/j.meatsci.2022.109043.
2. Wan, Z.; Wei, R.; Yang, M.; Xu, X.; Tian, X. Mechanism of brittleness deterioration of pork meatballs induced by freeze–thaw cycles based on ice crystals and molecular conformation. *Food Research International* **2025**, *202*, 115711, doi:10.1016/j.foodres.2025.115711.
3. You, Q.; Wang, Z.; Tian, X.; Xu, X. A multi-block data approach to assessing beef quality: ComDim analysis of hyperspectral imaging, 1H NMR, electronic nose and quality parameters data. *Food Chemistry* **2023**, *425*, 136469, doi:10.1016/j.foodchem.2023.136469.
